# Gut microbiota transfer from autoimmune dry eye mice imprints stereotypic B cell receptor repertoires in the lacrimal gland and induces disease

**DOI:** 10.3389/fimmu.2026.1827057

**Published:** 2026-06-16

**Authors:** Seonghwan Kim, Soobin Lee, Soyeon Ju, Jaewoong Bae, Jin Suk Ryu, Yerim Heo, Wan Jae Choi, Kum-Joo Shin, Seok-Jin Kim, Namphil Kim, Hansol Choi, Jiyun Park, Eunjae Lee, Chang Ho Yoon, Sunghoon Kwon, Junho Chung, Mee Kum Kim

**Affiliations:** 1 Department of Ophthalmology, Seoul National University College of Medicine, Seoul, Republic of Korea; 2 Department of Ophthalmology, Seoul Metropolitan Government Seoul National University Boramae Medical Center, Seoul, Republic of Korea; 3 Laboratory of Ocular Regenerative Medicine and Immunology, Biomedical Research Institute, Seoul National University Hospital, Seoul, Republic of Korea; 4 Department of Electrical and Computer Engineering, Seoul National University, Seoul, Republic of Korea; 5 Department of Biomedical Science, Seoul National University College of Medicine, Seoul, Republic of Korea; 6 Department of Biochemistry and Molecular Biology, Seoul National University College of Medicine, Seoul, Republic of Korea; 7 R&D Center, Hecto Healthcare Co., Ltd., Seoul, Republic of Korea; 8 Interdisciplinary Program in Bioengineering, Seoul National University, Seoul, Republic of Korea; 9 Bio-MAX Institute, Seoul National University, Seoul, Republic of Korea; 10 Cancer Research Institute, Seoul National University, Seoul, Republic of Korea; 11 Institute on Aging, Seoul National University, Seoul, Republic of Korea; 12 Transplantation Research Institute, Seoul National University College of Medicine, Seoul, Republic of Korea

**Keywords:** B cell receptor repertoire, fecal microbiota transplantation, gut-eye-immune axis, microbiome, Sjögren disease

## Abstract

Gut microbiota and humoral immunity have been suggested as key players in the pathogenesis of Sjögren disease (SjD), but their mechanisms remain unclear. In this study, we transferred the gut microbiota of SjD-like autoimmune dry eye disease model mice to B6 mice, then characterized the resulting gut microbiome composition, clinical ocular phenotype, and B cell receptor (BCR) repertoire. Notable changes were observed in the gut microbiome of NOD-FMT mice, accompanied by SjD-like clinical features, including elevated corneal fluorescein staining scores, reduced tear production, increased IL-6 mRNA levels, and decreased MUC5AC mRNA levels. Additionally, stereotypic B cell receptor (BCR) clonotypes were shared at significantly higher frequencies in NOD-FMT mice than in controls. The majority of B cell clones encoding these stereotypic clonotypes developed and expanded locally in the lacrimal gland, and some also achieved systemic presence. These results uncover a gut–ocular immune axis in which microbiota transfer induces stereotyped, systemically disseminating BCR clonotypes that contribute to the immunopathogenesis of autoimmune dry eye disease.

## 1 Introduction

Sjögren disease (SjD) (1) is a chronic autoimmune disease characterized by lymphocytic infiltration of the exocrine glands and multiple other organs, leading to sicca manifestations and a wide spectrum of extra-glandular involvement (2). SjD is one of the most prevalent systemic rheumatic autoimmune diseases (3, 4). The precise etiology of SjD remains elusive, but complex interactions among genetic, environmental, and immunological factors have been implicated (5). Treatment strategies targeting autoimmune pathways have been widely discussed (6–9), and numerous investigational agents are currently being tested in clinical trials (10). Given its chronicity and symptom burden, SjD demands the development of effective therapeutic approaches.

Since the launch of National Institutes of Health (NIH) Human Microbiome Project to explore interactions between humans and their microbiota in health and disease (11, 12), an increasing body of evidence has shown that gut microbiome dysbiosis is closely related to autoimmune diseases through the modulation of the innate or adaptive immune systems (13–17). Building on these insights, fecal microbiota transplantation (FMT) from healthy donors to patients has emerged as a potential therapeutic strategy for autoimmune conditions (18–22). Recent studies have highlighted association between gut dysbiosis and SjD, proposing that gut microbiota may contribute dry eye disease through the gut-eye-lacrimal gland (LG)-microbiome axis (23–26). Nevertheless, the precise role of gut dysbiosis in the pathogenesis of SjD, as well as the specific microbial taxa that may exacerbate or ameliorate dry eye syndrome, remains poorly understood.

Humoral immunity plays a central role in the development and progression of autoimmune diseases (27–29), and autoantibodies targeting exocrine glands are frequently detected in SjD patients (30, 31). The B cell receptor (BCR) repertoire, which enables B cells to recognize and bind to specific antigens (32), can vary substantially depending on the pathogenetic context of the autoimmune disease (33). However, the composition and pathogenic relevance of the BCR repertoire in SjD, particularly in relation to the microbiome, remain largely unexplored.

Here, we investigate the relationship between gut dysbiosis and ocular inflammation in SjD and examine associated changes in the BCR repertoire using a mouse model. Specifically, we performed FMT from an autoimmune dry eye mouse strain (NOD.B10.H2^b^) into C57BL/6 (B6) mice to access disease transfer, microbiome alterations, and BCR repertoire changes.

## 2 Materials and methods

### 2.1 Fecal preparation for FMT

Feces were collected from 30 male NOD.B10.H2^b^ mice over 20 weeks old (The Jackson Laboratory, Bar Harbor, ME, USA, RRID: IMSR_JAX:002591). The collected feces (1, 200 mg) were dissolved in 30 mL of phosphate-buffered saline (PBS) and centrifuged at 500-1, 000 g for 30 seconds. The supernatant was collected and stored at -80 °C until use.

### 2.2 FMT to B6 mice

**Figure 1:**
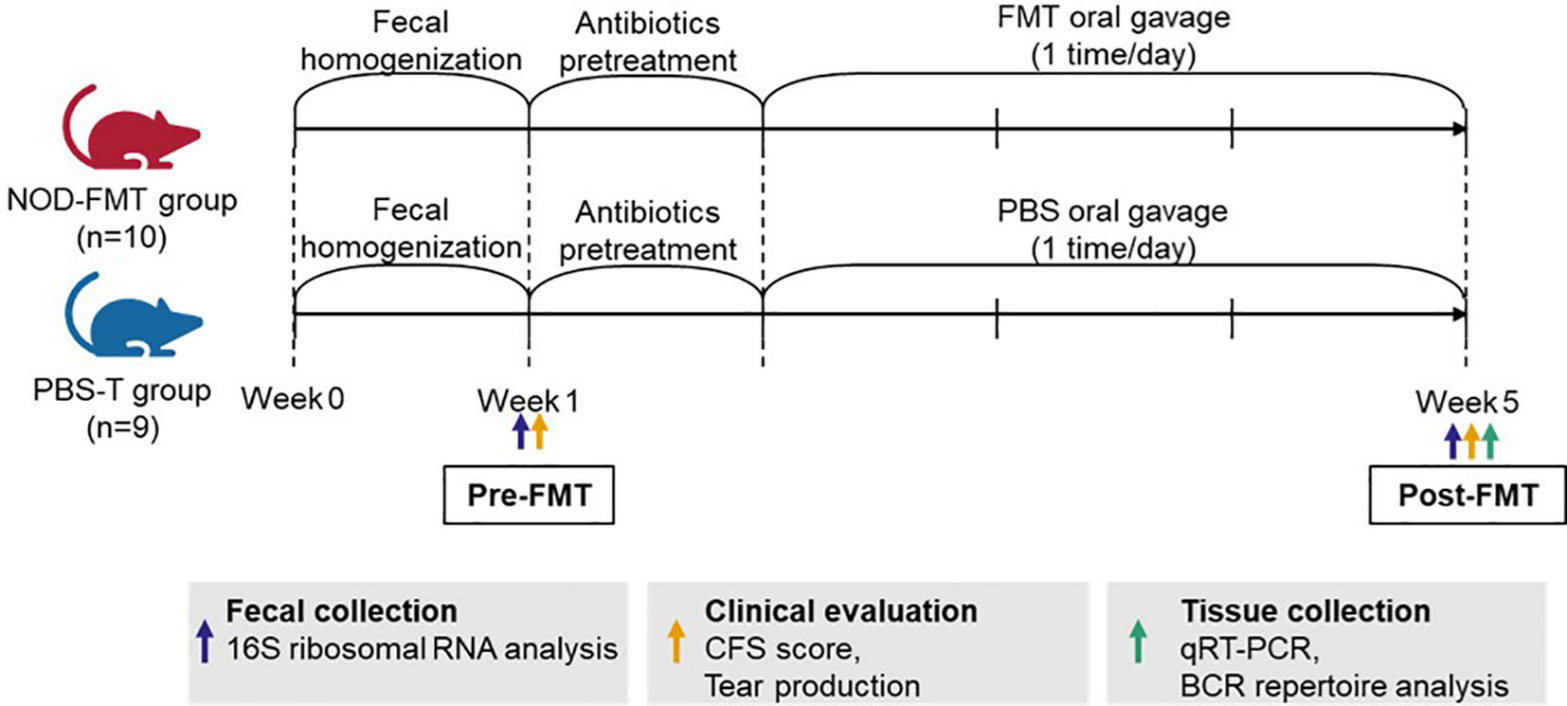
Scheme of the FMT experiment.

Prior to FMT, fecal homogenization was performed by mixing and redistributing mice feces into each cage for 7 days to minimize the differences in fecal microbiome composition among B6 mice (KOATECH Inc., Gyeonggi-do, Korea, RRID: MGI:3028467) at baseline (**Figure 1**). Afterwards, the mice were pretreated with antibiotics by adding a cocktail of 1 g/liter ampicillin, 500 mg/liter vancomycin, and 1 g/liter metronidazole (all from Sigma-Aldrich, St. Louis, MO, USA) to the drinking water for 7 days. For FMT, 200 μL of the stored supernatant was gavaged to each of ten 5-week-old B6 mice (NOD-FMT group) once a day for three weeks (**Figure 1**). In a parallel experiment, 200 μL of PBS was gavaged to nine mice (PBS-T group) following the same schedule.

### 2.3 Clinical evaluation

Corneal staining was performed by instilling one drop of 0.25% fluorescein dye to the lower conjunctival sac, incubating for 30 sec, then gently washing with 1 mL of normal saline. CFS scores were determined by three independent investigators in a blind manner, using a microscope (Olympus Compact Stereo Microscope SZ61, RRID: SCR_018950; Olympus Corporation, Tokyo, Japan) under cobalt blue illumination with the National Eye Institute (NEI) scale (0–15) (34). The median value was used for statistical analysis.

Tear production was measured using phenol red-impregnated cotton threads (Zone-Quick, Showa Yakuhin Kako, Tokyo, Japan). After placing the threads to the lateral canthus for 60 sec, the wet length of the thread was measured in millimeters (mm). Body weights of the mice were also measured immediately after tear production measurements.

### 2.4 Quantitative real-time PCR

The corneal and conjunctival tissues were harvested from the NOD-FMT, and PBS-T mice at the point of sacrifice (**Figure 1**). These tissues were then cut into small pieces and lysed in RNA isolation reagent (RNeasy mini kit, Qiagen, Venlo, Netherlands). After sonication with a probe sonicator (Ultrasonic Processor, Cole Parmer Instruments, Vernon Hills, IL, USA), total RNA was extracted using the RNeasy Mini kit (Qiagen), and first‐strand cDNA was synthesized by reverse transcription with the High-Capacity RNA‐to‐cDNA Kit (Applied Biosystems, Foster City, CA, USA). The qRT-PCR was conducted as previously described (35). Real‐time amplification was performed using the TaqMan Universal PCR Master Mix (Applied Biosystems) in an automated instrument (ABI 7500 Real Time PCR System, RRID: SCR_019334) targeting tumor necrosis factor (TNF)-α (Mm00443258_m1, Thermo fisher, Waltham, MA, USA), interferon (IFN)-γ (Mm01168134_m1, Thermo fisher), interleukin (IL)-1β (Mm00434228_m1, Thermo fisher), IL-6 (Mm00446190_m1, Thermo fisher), IL-10 (Mm00439614_m1, Thermo fisher), transforming growth factor (TGF)-β (Mm01178820_m1, Thermo fisher), epidermal growth factor (EGF) (Mm00438696_m1, Thermo fisher), and mucin 5AC (MUC5AC) (Mm01276718_m1, Thermo fisher). The relative levels of mRNA species were calculated by using the average value of the PBS-T group as a calibrator, to which a value of one was assigned.

### 2.5 Amplification and sequencing of 16S rRNA of fecal microbiota

Before and after the FMT administration (**Figure 1**), namely Pre-FMT and Post-FMT, respectively, fecal pellets were collected from the anus of each mouse by holding it and allowing defecation. The collected feces were immediately frozen and stored at -80 °C. Bacterial genomic DNA was extracted from the collected fecal samples using the Maxwell^®^ RSC PureFood GMO and Authentication Kit (Promega, Medison, WI, USA), following the manufacturer’s instructions. The extracted DNA was quantified using a QuantiFluor^®^ ONE dsDNA System. All extracted DNA samples were stored at -20 °C. The microbiota composition of the mouse was analyzed by 16S rRNA amplicon sequencing using Illumina Miseq (Illumina, Inc., San Diego, CA, USA, Illumina MiSeq System, RRID: SCR_016379), in which the V3-V4 region of the bacterial 16S rRNA gene was amplified using a primer set following the manufacturer’s protocol. The primer set consisted of Forward primer (5’ to 3’ sequence: TCGTCGGCAGCGTAGATGTGTAAGAGACGCCTACGGGNGGCWGCAG) and Reverse primer (5’ to 3’ sequence: GTCTCGTGGGCTCGGAGATGTGTATAAGAGACAGGACTACHVGGGTATCTAATCC).

### 2.6 Data processing for analysis of 16S rRNA sequencing data

The sequencing data was processed and analyzed using plugins from the QIIME2 (ver.2023.05, RRID: SCR_021258) package as described previously (36). Once the data artifact had been created for use in QIIME2, the forward and reverse Illumina adapters were identified and removed from the demultiplexed samples using the “cutadapt trim-paired” function. Paired-end sequences were merged using the “vsearch merge-pairs” function, and bases with poor quality scores were filtered using the “quality-filter q-score” function. Denoising was performed using the Deblur algorithm, with a trim-length of 251. Then the sequences were aligned against the SILVA database (“silva-138.1-99-nb-classifier.qza”, https://www.arb-silva.de/archive/release_138.1/Exports, RRID: SCR_006423) for the taxonomic annotation (i.e., phylum, class, order, family, genus and species) as described previously (36). Bacterial sequences were filtered by using the taxa plugin to remove archaea, eukaryotes, mitochondrial sequences, and chloroplast genomes. Samples with a total frequency of 0 were removed. For the purpose of sampling depth normalization, the “feature-table rarefy” function was used to rarefy the samples. Following this, the taxon abundance by level was obtained by using the “taxa collapse function”, with identity thresholds set to 97% sequence similarity for genus-level assignment. Abundance was calculated using the “feature-table relative-frequency” function, in which the frequency of each taxon in the sample was divided by the total sum of frequencies within the sample.

Alpha diversity was assessed using the Shannon diversity index, and beta diversity was calculated based on Bray-Curtis dissimilarity using QIIME2’s diversity plugin. Beta diversity metrics were visualized via principal coordinate analysis (PCoA). Differential abundance analysis between groups was performed using both Analysis of compositions of microbiomes with bias correction (ANCOM-BC2, RRID: SCR_024901) and the Mann-Whitney U test (37).

### 2.7 High throughput sequencing of BCR heavy chain

The spleen and LG were harvested from the mice at the point of sacrifice, and peripheral blood (PB) was collected by heart puncture and pooled by each group. Peripheral blood mononuclear cells (PBMCs) were isolated by density gradient centrifugation using Ficoll Paque (Cytiva, Marlborough, MA, USA), according to the manufacturer’s protocol. Total RNA was extracted using TRIzol reagent (Invitrogen, Carlsbad, CA, USA), following the manufacturer’s protocol. Genes encoding the variable domain of the BCR heavy chain (VH) and a part of the first constant domain (CH1) were amplified as described previously with adequate modifications (38). Primer sequences are listed in **Supplementary Table 1**.

Total RNA was reverse-transcribed by CH1 domain gene-specific primers containing unique molecular identifier (UMI) and the reverse Illumina adaptor sequences, using the SuperScript™ IV First-Strand Synthesis System (Invitrogen). The synthesized complementary DNA (cDNA) was purified using 1.8x volume of SPRI beads (AMPure XP, Beckman Coulter, Brea, CA, USA) and eluted in 35 μL of DW. The purified cDNA was then subjected to second-strand synthesis with VH gene-specific primers containing the forward Illumina adaptor sequences, using KAPA biosystems (KAPA HiFi HotStart, Roche, Basel, Switzerland). The thermal conditions were as follows: 95 °C for 5 min, 98 °C for 1 min, 60 °C for 45 sec, and 72 °C for 5 min. The synthesized double-strand DNA (dsDNA) was purified using 1x volume of SPRI beads then indexed PCR amplification was performed by forward and reverse Illumina adaptor primers containing index sequences. The PCR conditions were as follows: 95 °C for 5 min; 25 cycles of 98 °C for 30 sec, 65 °C for 30 sec, and 72 °C for 1 min; and 72 °C for 5 min. The PCR products were subjected to gel electrophoresis in 1.5% agarose gel, size-selected using QIAquick Gel Extraction Kit (Qiagen), and purified by 1x volume of SPRI beads.

The quality and concentration of the final BCR heavy chain libraires were determined by the Agilent 4200 TapeStation System (RRID: SCR_018435) (G2991A, Agilent technologies, Santa Clara, CA, USA) and sequenced on the Illumina NovaSeq 6000 Sequencing System (RRID: SCR_016387) in 250PE mode (SP flow cell, Illumina Inc.).

### 2.8 Construction of the BCR repertoire

Raw paired-end reads were merged using PEAR v0.9.10 (RRID: SCR_003776) in default settings, then quality-filtered using the condition q20p95, retaining reads with a Phred score of 20 or higher in over 95% of base pairs as previously described (38). The products were clustered based on UMI sequences, using Clustal Omega v.1.2.4 (RRID: SCR_001591), excluding singletons in order to extract consensus sequences (38). Isotypes were annotated by aligning constant regions with the IMGT - the international ImMunoGeneTics information system (RRID: SCR_012780) constant gene database (39). V(D)J regions were annotated using IgBLAST v.1.17.1 (RRID: SCR_002873), with reads aligned to IGHV, IGHJ, and CDR1/2/3 regions (40). Unannotated reads were considered nonfunctional and excluded from analysis, and the remaining reads were identified as productive BCR sequences.

Following the annotation processes, BCR sequences were grouped into clonotypes, which were defined as a group of BCR sequences sharing the same IGHV/J gene pairs and possessing identical HCDR3 amino acid sequences. To eliminate potential cross-contamination among individual samples, BCR sequences with identical nucleotide sequences found in more than two mice were removed. The removed sequence reads constituted 0.26% (7, 369 out of 2, 878, 417), 2.14% (249, 402 out of 11, 666, 610) and 2.06% (9, 305 out of 452, 019) of all BCR sequences found in LG, spleen, and PB, respectively.

### 2.9 Quantification of similarities between BCR repertoires

Similarities between BCR repertoires were calculated between all mouse pairs based on vectors representing the composition of BCR clonotypes in each mouse. These vectors were created by designating the presence of individual clonotypes in the union of clonotypes from two mice as 1s, and absence as 0s. The similarity of BCR repertoires between two mice was determined as the cosine similarity of those vectors.

### 2.10 Construction of single-chain variable fragment phage display library

A scFv phage display library was constructed using the total RNA isolated from the spleens of 10 NOD-FMT mice and the primers listed in **Supplementary Table 16**, as previously described (41). Briefly, reverse transcription was performed using the SuperScript™ IV First-Strand Synthesis System (Invitrogen) with Oligo(dT)20 primer (Invitrogen). The synthesized cDNA was subjected to second-strand synthesis and amplification using V- and J-gene-specific primers targeting VH, kappa light chain variable domain (Vκ), and lambda light chain variable domain (Vλ). The PCR conditions were as follows: 95 °C for 5 min; 25 cycles of 98 °C for 30 sec, 60 °C for 30 sec, and 72 °C for 1 min; and 72 °C for 5 min. The PCR products were separated on a 1.5% agarose gel, size-selected using the QIAquick Gel Extraction Kit (Qiagen) and quantified using a Thermo Fisher NanoDrop One/OneC Microvolume UV-Vis Spectrophotometer (RRID: SCR_023005). Subsequently, 500 ng of VH, 250 ng of Vκ, and 250 ng of Vλ products were mixed and subjected to overlap-extension PCR to generate scFv genes. The PCR conditions were as follows: 95 °C for 5 min; 25 cycles of 98 °C for 20 sec, 65 °C for 15 sec, and 72 °C for 1 min; and 72 °C for 10 min. The amplified scFv gene was gel-purified and cloned into the pComb3XSS phagemid vector (RRID: Addgene_63890) (42), which contains 6xHis-HA tag sequence. The resulting library was transformed into *E.coli* ER2738 cells, and phage display library was generated by using VCSM13 helper phage as previously described (42). Briefly, the library was transformed into ER2738 by electroporation and incubated in super broth (SB) medium (43) at 37 °C with shaking. Subsequently, 50 μg/mL carbenicillin and 1x10^12^ plaque forming unit (PFU) of VCSM13 helper phage were added (44). After 2 h of incubation in a 37 °C shaker, 70 μg/mL of kanamycin was added and the culture was incubated overnight at 37 °C with shaking. The next day, the culture was centrifuged and the supernatant was incubated with 4% (w/v) polyethylene glycol (PEG) and 3% (w/v) NaCl on ice for 30 min, then centrifuged at 10, 000 rpm to pellet the phage. The phage pellet was resuspended in 1% (w/v) BSA in Tris-buffered saline (TBS) and subjected to biopanning.

### 2.11 Biopanning and enzyme-linked immunosorbent assay

PmE-L and mouse IgG (Invitrogen, I8765) were conjugated to M-270 Epoxy Dynabeads (Invitrogen, 14301) according to the manufacturer’s instructions. The phage display library was subjected to biopanning to PmE-L - and mouse IgG - conjugated M-270 Epoxy beads (Invitrogen) as previously described (43). The PmE-L peptide (VGIRYTGCSVNPARSFGC) (45) was synthesized by Peptron (Daejeon, Korea).

After four rounds of biopanning, PmE-L- and mouse IgG-reactive clones were screened by ELISA using bacterial periplasmic extracts, as previously described (46). Briefly, individual *E.coli* colonies from the third and fourth rounds of biopanning were inoculated into SB containing 50 μg/mL carbenicillin in 96-well Round Bottom Deep well plates (Axygen, P-2ML-SQ-C). When the optical density at 600 nm (OD_600_) reached 0.6 - 0.7, protein expression was induced with 1mM isopropyl β-D-1-thiogalactopyranoside (IPTG) (47) and the cultures were incubated overnight at 18 °C with shaking. The bacteria were pelleted by centrifugation and resuspended with 88 μL of TES extraction buffer (50mM Tris-HCl, 1mM EDTA, 20% (w/v) sucrose, pH 8.0) followed by incubation on ice for 30 min. Subsequently, 132 μL of hypotonic TES buffer (diluted 1:10 in DW) was added, and the samples were incubated on ice for additional 30 min to cause osmotic shock and lyse the bacterial outer membrane (46, 48). The samples were then centrifuged, and the supernatants were collected.

For ELISA, wells of microtiter plates were coated with either PmE-L (5μg/ml in PBS) or mouse IgG (2.5μg/ml in PBS) at 4 °C overnight, then blocked with 1% (w/v) BSA in PBS or 3% (w/v) BSA in PBS, respectively, as previously described (49). The bacterial periplasmic extract was added to the wells, incubated for 2 h at 37 °C then washed three times with 0.05% (v/v) PBS-Tween.

The amount of bound antibody was determined using horseradish peroxidase (HRP) conjugated-anti-HA antibody (Roche Cat# 12013819001, RRID: AB390917) and ABTS substrate solution (Thermo Fisher Scientific, 002024). The absorbance was measured at 405 nm using Multiskan Sky microplate reader (Thermofisher Scientific). Background signals were measured from BSA-blocked wells without antigen coating. The nucleotide sequences of antigen-reactive scFv clones were determined by Sanger sequencing as previously described (49).

### 2.12 Statistical analyses

Prior to statistical analysis, normality of the data distribution was assessed using the Shapiro–Wilk test (50). Paired differences in CFS scores between groups were evaluated using the Wilcoxon signed-rank test with the Pratt zero method to account for zero-difference observations. Comparisons of tear production adjusted by body weight and qRT-PCR analysis were performed with the paired t-test. Clinically evaluated features and qRT-PCR analysis results are presented as mean ± SEM.

Beta diversity was assessed using permutational multivariate analysis of variance (PERMANOVA) with 10, 000 permutations based on Bray-Curtis dissimilarity matrices, implemented via the adonis function in the vegan package (51). Differences in taxonomic composition between groups were evaluated using two approaches: ANCOM-BC and the Mann-Whitney test. For ANCOM-BC, taxa with a p-value < 0.05 and an absolute effect size (LFC) > 2 were considered significant. The Mann-Whitney test was applied to genera with relative abundance > 1% in at least one group. All statistical tests were performed using the rstatix package (v0.7.2, RRID: SCR_021240) in R (v4.3.0, RRID: SCR_001905), and figures were generated using ggplot2 (v3.4.4, RRID: SCR_014601).

Clonotype similarities between two groups and the degree of sharing of stereotypic clonotypes within each group were compared using the Mann-Whitney test. The Mann-Whitney test was also used to compare the mean frequency of all clonotypes and stereotypic clonotypes across the groups. The number of LG or spleen stereotypic clonotypes found in vice versa organs or PB was assessed using the chi-square test. In consideration of the substantial sample size, the Cramer V test, ranging from 0 to 1, was utilized to identify practically meaningful relationships, with larger values indicating stronger associations between the variables (52). Statistical analysis of the BCR repertoire was performed in Python (v3.6.13; RRID: SCR_008357) using the SciPy 1.5.4 package (RRID: SCR_008058). In all statistical analyses, significance was defined as: *p < 0.05, **p < 0.01, ***p < 0.001, ****p < 0.0001.

## 3 Results

### 3.1 FMT from an autoimmune dry eye mouse strain induces lacrimal gland inflammation and tear loss

**Figure 2:**
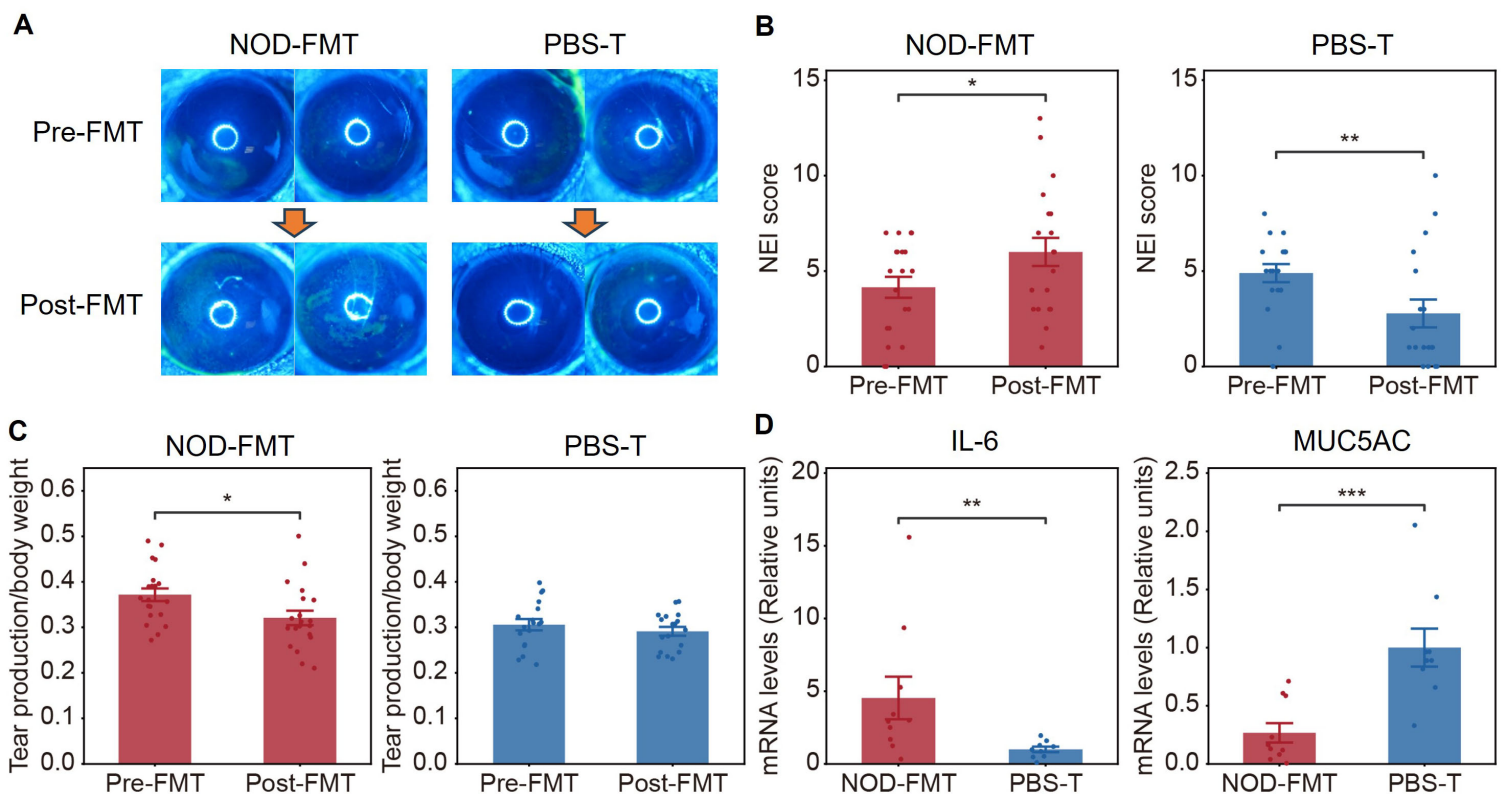
Clinicopathologic features of dry eye disease conveyed to B6 mice after FMT. **(A)** Representative photographs of the corneal epithelia of B6 mice subjected to FMT from NOD.B10.H2^b^ mice (NOD-FMT) and the control group with PBS transfer (PBS-T). **(B)** Changes in corneal fluorescein staining (CFS) scores determined by the National Eye Institute (NEI) scale (0-15). **(C)** Changes in tear production adjusted by body weight (mm/gram). **(D)** mRNA levels of IL-6 and MUC5AC in corneal and conjunctival tissues determined by qRT-PCR. The values are shown as relative units, calculated by designating the average level in the PBS-T group as the value of one. The data are presented in mean values ± standard error of mean (SEM) **(B–D)**, where each symbol indicates data from an individual mouse. *p<0.05, **p<0.01, ***p<0.001, as analyzed by Wilcoxon signed-rank test **(B)**, paired T-test **(C)**, and Mann-Whitney test **(D)**.

Feces were collected from 30 NOD.B10.H2^b^ mice older than 20 weeks exhibiting autoimmune dry eye disease, and their microbiomes were transferred to ten B6 mice (NOD-FMT group). In parallel, nine B6 mice received oral PBS gavage (PBS-T group) (**Figure 1**). In the NOD-FMT group, NEI scale, measured by corneal fluorescein staining (CFS), increased significantly post-FMT, relative to baseline (p=0.046, Wilcoxon Test, **Figures 2A, B**). Tear production adjusted by body weight (mm/gram) decreased significantly in the NOD-FMT group (p=0.024, paired t-test, **Figure 2C**), whereas no changes were observed in the PBS-T group. qRT-PCR analysis on TNF-α, IFN-γ, IL-1β, IL-6, IL-10, TGF-β, EGF and MUC5AC mRNA levels in the collected corneal and conjunctival tissues and LG tissues revealed elevated IL-6 mRNA and reduced MUC5AC mRNA levels in the NOD-FMT group compared to the PBS-T group (p=0.004 and 0.0005, respectively, Mann-Whitney test) (**Figure 2D**) indicating tissue inflammation and impaired mucin production. No statistically significant differences were observed in the expression levels of TNF-α, IFN-γ, IL-1β, IL-10, TGF-β, or EGF between the groups; these data are presented in **Supplementary Figure 1**.

### 3.2 FMT alters gut microbial composition.

**Figure 3:**
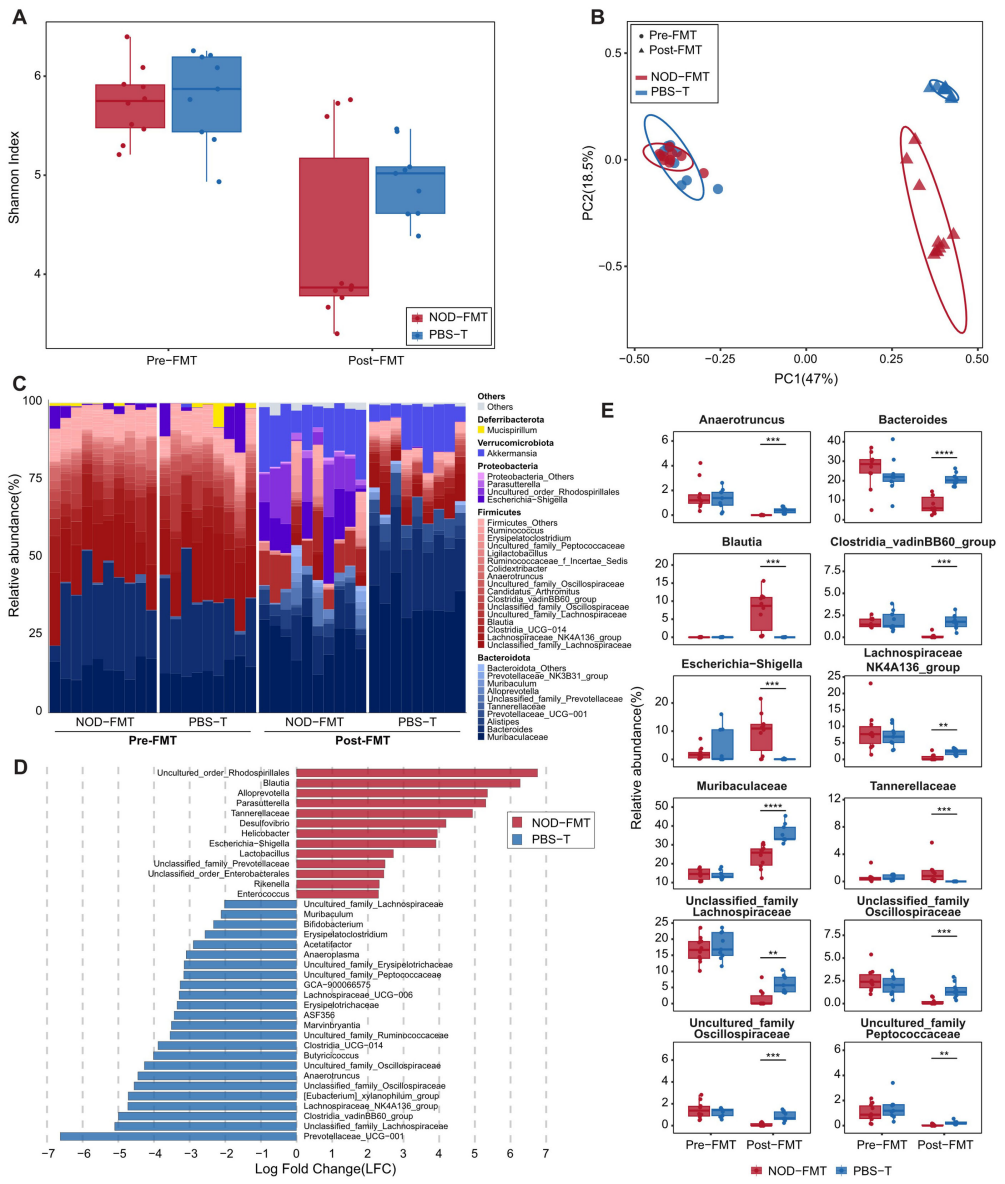
Alterations in a- and b-diversities and microbiota composition in B6 mice after FMT. **(A)** a-diversity in NOD-FMT group and the PBS-T group at the Pre-FMT and Post-FMT timepoints, measured using the Shannon index. **(B)** Principal coordinates analysis (PCoA) at the amplicon sequence variant (ASV) level. The axes indicate the proportion of variation in the overall community dissimilarity explained by each principal coordinate. Statistical significance of group differences was assessed using PERMANOVA (p=0.0004). **(C)** The stacked bar graph demonstrates proportional distributions of the microbiota in each sample. Each genus is represented by a colored segment within each bar. Colors are grouped by phylum, with darker shades indicating the most dominant genus within each phylum, and lighter shades representing less abundant genera from the same phylum. ‘Others’ refer to genera belonging to all phyla, with <1% abundance at the phylum level. The ‘[phylum]_Others’ refer to genera belonging to phyla with >1% abundance at the phylum level, but less than 1% at the genus level. **(D)** Differential abundance analysis using ANCOM-BC between the NOD-FMT and PBS-T groups at the Post-FMT timepoint. Taxa with LFC scores of > 2 and a p-value of <0.05 were considered significant. **(E)** Relative abundances of selected genera showing significant differences between groups. Only genera with >1% abundance in at least one group and significant post-FMT group-wise differences (Wilcoxon test) are shown. Relative abundance was calculated by dividing the frequency of each taxon in the sample by the total sum of frequencies in the same sample. **p <0.01, ***p <0.001, ****p <0.0001.

Fecal microbiota profiles were analyzed by 16S rRNA amplicon sequencing. At baseline (Pre-FMT point), Alpha diversity (Shannon index) and Beta diversity (Bray-Curtis dissimilarity index) did not differ significantly between the two groups, consistent with successful fecal homogenization (**Figures 3A, B**). After FMT (Post-FMT point), beta diversity diverged markedly between PBS-T and NOD-FMT group (**Figure 3B**) with greater taxonomic differences (**Figure 3C**). ANCOM-BC analysis identified significantly altered taxa (log fold change, LFC >2.0, p < 0.05) including increased *Uncultured_order_Rhodospirillales, Blautia, Alloprevotella, Parasutterella, Tannerellaceae, Desulfovibrio, Helicobacter, Escherichia-Shigella, Lactobacillus, Unclassified_family_Prevotellaceae, Unclassified_order_Enterobacterales*, and *Rikenella*, in the NOD-FMT group compared to PBS-T group (**Figure 3D**; **Supplementary Table 2**).

Genus-level analysis confirmed upregulation of *Blautia, Escherichia-Shigella*, and *Tannerellaceae* (p = 1.6 × 10^-4^, 9.68 × 10^-4^, and 2.4 × 10^-3^, respectively), and downregulation of *Anaerotruncus* (p = 1.22 × 10^-4^), *Bacteroides* (p = 2.17 × 10^-5^), *Clostridia_vadinBB60_group* (p = 2.7 × 10^-4^), *Lachnospiraceae_NK4A136_group* (p = 3.87 × 10^-3^), *Unclassified_family_Lachnospiraceae* (p = 4.7 × 10^-3^), *Muribaculaceae* (p = 4.33 × 10^-5^), *Unclassified_family_Oscillospiraceae* (p = 6.96 × 10^-4^), *Uncultured_family_Oscillospiraceae* (p = 3.73 × 10^-4^), and *Uncultured_family_Peptococcaceae* (p = 2.94 × 10^-3^) in the NOD-FMT group compared to PBS-T group (**Figure 3E**).

### 3.3 FMT shapes concordant BCR repertoires

BCR repertoires were generated from the LG and spleen tissues at the Post-FMT point. Clonotype were defined as BCR sequences with identical IGHV/J gene usage and CDR3 amino acid sequences. LG BCR repertoires contained 12, 249 and 22, 691 BCR clonotypes in NOD-FMT and PBS-T groups, respectively, while spleen BCR repertoires contained 1, 778, 376 and 925, 445 BCR clonotypes. Additional untreated B6 mice (n=5) were included as BCR repertoire controls; LG and spleen BCR repertoires contained 3, 956 and 687, 306 BCR clonotypes, respectively (**Supplementary Tables 3**, **4**).

**Figure 4:**
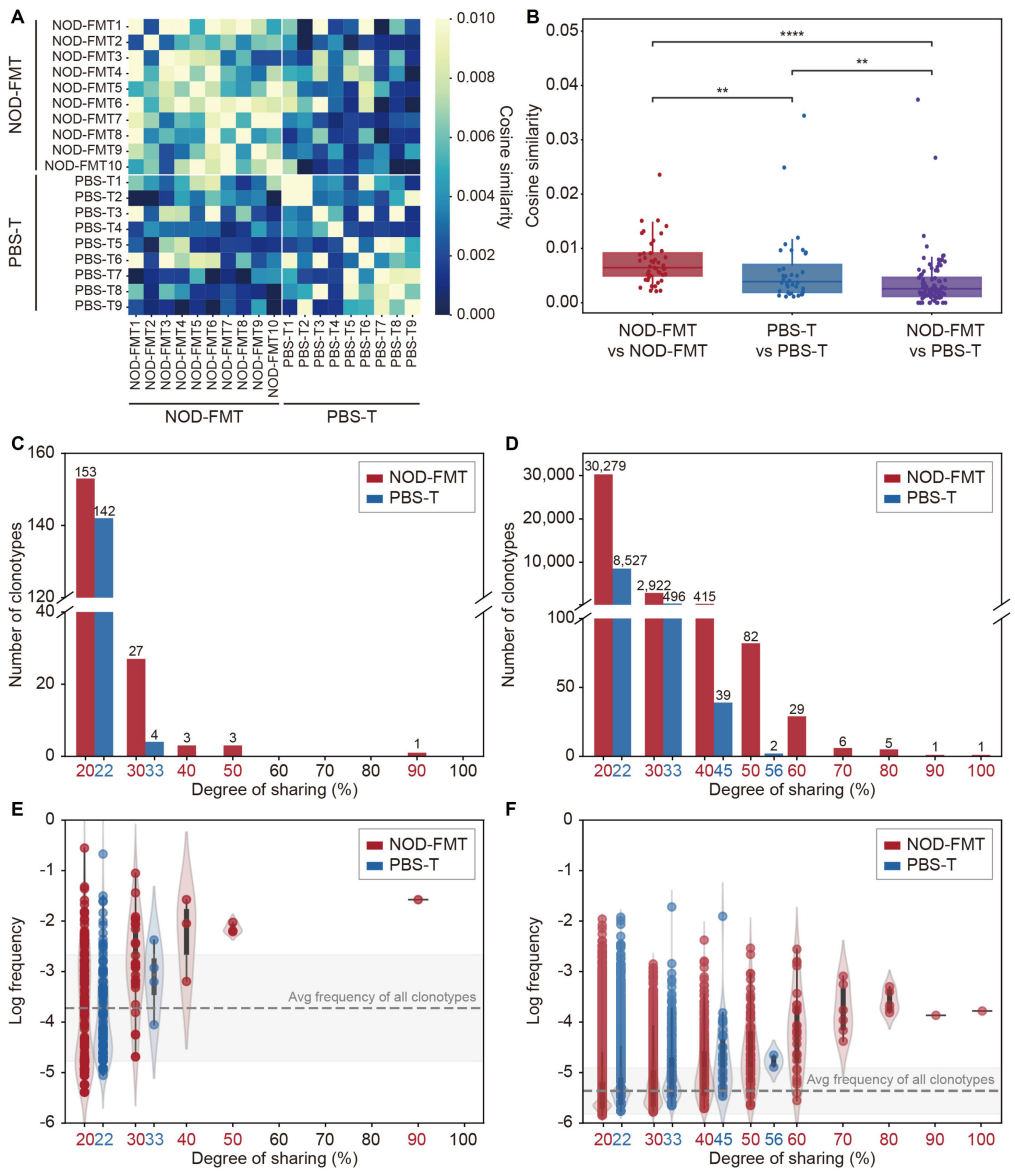
Stereotypic BCR clonotypes in the lacrimal gland and spleen repertoire. **(A)** Heatmap of clonotype similarity between mice from NOD-FMT and PBST groups. **(B)** Clonotype similarity among mouse pairs within NOD-FMT and PBS-T groups. **(C–F)** The number of stereotypic clonotypes in the BCR repertoires of the **(C)** LG or **(D)** spleen according to the degree of sharing, which is defined as the percentage of stereotypic clonotype-harboring mice out of ten NOD-FMT mice or nine PBS-T mice. Log frequency of stereotypic clonotypes in the BCR repertoires of the **(E)** LG or **(F)** spleen, according to the degree of sharing. The gray line represents the average frequency of all BCR clonotypes in the NOD-FMT or PBS-T groups, and the shaded areas indicate standard deviation of the frequency of all clonotypes (E, F). **p < 0.01, ****p < 0.0001.

Pairwise BCR similarity was computed for all possible mouse pairs within each group (NOD-FMT, n = 10; PBS-T, n = 9) and across groups. Similarity analysis revealed that cosine similarity was significantly higher within NOD-FMT group compared to PBS-T or cross-group comparisons (p=0.001 or p<0.0001, Mann-Whitney test, **Figures 4A, B**). Thus, FMT shaped concordant BCR repertoires distinct from PBS-T group.

Group-specific clonotypes were identified after excluding those shared with controls and the other experimental group. In the LG of the NOD-FMT group, 1, 3, 3, 27, and 153 clonotypes were shared among 90% (9/10), 50% (5/10), 40% (4/10), 30% (3/10) and 20% (2/10) of the total 10 mice, respectively (**Figure 4C**). By contrast, in the LG of PBS-T group, only four and 142 clonotypes were shared by 33% (3/9) and 22% (2/9) of the total 9 mice, respectively (**Figure 4C**), representing a significant discrepancy (p<0.0001, Mann-Whitney test, **Supplementary Table 5**).

A similar trend was observed in the spleen. In the NOD-FMT group, 1, 1, 5, 6, 29, 82, 415, 2, 922, and 30, 279 clonotypes were shared by 100% (10/10), 90% (9/10), 80% (8/10), 70% (7/10), 60% (6/10), 50% (5/10), 40% (4/10), 30% (3/10) and 20% (2/10) of mice (**Figure 4D**). In contrast, PBS-T group exhibited only 2, 39, 496 and 8, 527 clonotypes were shared by 56% (5/9), 45% (4/9), 33% (3/9) and 22% (2/9), respectively (**Figure 4D**). This again represented a significant discrepancy (p<0.0001, Mann-Whitney test, **Supplementary Table 5**).

Taken together, these analyses demonstrated that FMT induced the emergence of highly concordant BCR repertoires in NOD-FMT group, both in the LG and systemically.

### 3.4 FMT drives preferential expansion of stereotypic BCR clonotypes

In LG, the average frequency of 187 stereotypic BCR clonotypes in NOD-FMT group (4.78 x 10^–3^) was significantly higher than that of all BCR clonotypes in the same group (8.05 x 10^–4^) (p<0.0001, Mann-Whitney test, **Supplementary Table 6**), representing top 3.28% of clonotypes. Frequency increased with the stereotypy (**Figure 4E**). In spleen, stereotypic clonotypes also expanded, though at lower frequencies (1.52 x 10–^5^ vs 4.69x10^–6^) with statistical significance (p<0.0001, Mann-Whitney test, **Figure 4F**; **Supplementary Table 6**). These data demonstrated preferential expansion of stereotypic clonotypes following FMT.

### 3.5 Stereotypic BCR clonotypes evolve locally and disseminate systemically

Germline-containing clonotypes were defined as clonotypes containing at least one BCR sequence with zero somatic hypermutation (SHM = 0). In NOD-FMT mice, the fraction of germline-containing clonotypes among LG stereotypic BCR clonotypes was 84%, which was significantly higher than that observed in the PBS-T group (61%) (p<0.0001, chi-square test, **Table 1**; **Supplementary Table 7**). In contrast, the fraction of germline-containing clonotypes among spleen stereotypic BCR clonotypes in NOD-FMT mice was 99.5%, comparable to that of the PBS-T group (99.5%) (p=0.708, chi-square test, **Table 2**; **Supplementary Table 8**). These results suggest that stereotypic BCR clonotypes detected in the LG following FMT are more likely to contain germline BCR sequences, indicating ongoing local generation of stereotypic clonotypes within the LG.

**Table 1:** Number of germline-containing clonotypes among LG stereotypic BCR clonotypes.

Group	Degree of sharing									Total
	2	3	4	5	6	7	8	9	10	
NOD-FMT	125/153 (81.7%)	25/27 (92.6%)	3/3 (100%)	3/3 (100%)	–	–	–	1/1 (100%)	–	157/187 (84.0%)
PBS-T	86/142 (60.6%)	3/4 (75.0%)	–	–	–	–	–	–	**-**	89/146 (61.0%)

**Table 2:** Number of germline-containing clonotypes among spleen stereotypic BCR clonotypes.

Group	Degree of sharing									Total
	2	3	4	5	6	7	8	9	10	
NOD-FMT	30161/30279 (99.6%)	2894/2922 (99.0%)	406/415 (97.8%)	72/82 (87.8%)	24/29 (82.8%)	4/6 (66.7%)	5/5 (100.0%)	0/1 (0.0%)	1/1 (100%)	33567/33740 (99.5%)
PBS-T	8482/8527 (99.5%)	491/496 (99.0%)	39/39 (100%)	2/2 (100%)	–	–	–	–	–	9014/9064 (99.5%)

To assess whether these locally enriched stereotypic clonotypes subsequently disseminate to peripheral compartments, we next examined clonotype overlap across tissues. In NOD-FMT mice, 65.8% of LG stereotypic BCR clonotypes were also found in spleen repertoires, which was significantly higher than 24.7% observed in the PBS-T group (p<0.0001, chi-square test, **Table 3**; **Supplementary Table 9**). By contrast, only 0.8% of spleen stereotypic BCR clonotypes were detected in LG BCR repertoires, which was similar to 1.0% of the PBS-T group (**Table 4**; **Supplementary Table 10**).

**Table 3:** Number of LG stereotypic BCR clonotypes found in the spleen of the same mouse.

Group	Degree of sharing									Total	
	2	3	4	5	6	7	8	9	10		
NOD-FMT	91/153 (59.5%)	25/27 (92.6%)	3/3 (100%)	3/3 (100%)	–	–	–	1/1 (100%)	–	123/187 (65.8%)
PBS-T	34/142 (23.9%)	2/4 (50.0%)	–	–	–	–	–	–	**-**	36/146 (24.7%)

**Table 4:** Number of spleen stereotypic BCR clonotypes found in the LG of the same mouse.

Group	Degree of sharing									Total
	2	3	4	5	6	7	8	9	10	
NOD-FMT	185/30279 (0.6%)	49/2922 (1.7%)	23/415 (5.5%)	12/82 (14.6%)	10/29 (34.5%)	3/6 (50.0%)	0/5 (0.0%)	1/1 (100%)	1/1 (100%)	284/33740 (0.8%)
PBS-T	75/8527 (0.9%)	12/496 (2.4%)	2/39 (5.1%)	1/2 (50%)	–	–	–	–	–	90/9064 (1.0%)

PB repertoires were further analyzed by pooling samples within each group, yielding 202, 436 clonotypes in the NOD-FMT group and 168, 763 clonotypes in the PBS-T group (**Supplementary Table 11**). In NOD-FMT group, 28.3% of LG stereotypic BCR clonotypes were also present in PB BCR repertoire, which was higher than the 18.5% of PBS-T group (p=0.050, chi-square test, **Supplementary Tables 12**, **13**). All high-stereotypy LG clonotypes (≥ 5 out of 10 NOD-FMT mice) were consistently detected across LG, spleen, and PB. In contrast, spleen stereotypic BCR clonotypes of NOD-FMT group showed limited dissemination to PB (6.5%), comparable to PBS-T group (5.8%) (p=0.065, chi-square test, **Supplementary Tables 14**, **15**).

Collectively, these findings indicate that stereotypic clonotypes preferentially evolve in the LG following FMT and subsequently disseminate systemically.

### 3.6 NOD-FMT induces autoreactive BCR clonotypes targeting AQP-5 and IgG

To directly assess whether FMT from NOD.B10.H2b mice induces autoreactive BCR responses in B6 recipient mice, we generated scFv phage display libraries using cDNA prepared from the spleens of NOD-FMT mice and performed biopanning against two antigens: PmE-L and mouse IgG.

PmE-L is a peptide derived from the aquaporin (AQP) of *Prevotella melaninogenica* that is homologous to human AQP-5 — a key water channel autoantigen in SjD. We previously reported that immunization with this peptide induced anti-AQP-5 antibodies and reduced salivary flow in mice (41, 44). AQP-5 is a water-channel protein expressed in salivary and lacrimal glands (53) and has been proposed as a potential autoimmune target in SjD, supported by abnormal distribution of AQP-5 in salivary gland biopsies (54), and elevated levels of anti-AQP-5 antibodies in the sera reported from SjD patients (55). In addition, the AQP homolog of *Prevotella melaninogenica* shares high sequence and structural similarity with human and mouse AQP-5 (41, 56), suggesting that molecular mimicry may contribute to the induction of anti-AQP-5 autoantibodies. Autoantibodies that bind the Fc portion of IgG, known as rheumatoid factor (RF), are commonly detected in SjD (57) and in mouse models mimicking SjD (58, 59). Therefore, mouse IgG was selected as the second antigen for biopanning. After biopanning, eight clones reactive to PmE-L and nine clones reactive to mouse IgG were selected by ELISA using periplasmic extracts of the bacterial cells (**Supplementary Figures 2A, B**).

In the LG BCR repertoire of both NOD-FMT and PBS-T group, one PmE-L–reactive clonotype (P_2G10) was found in two mice, but its mean frequency in NOD-FMT mice was over 600-fold higher than in PBS-T mice (**Supplementary Figures 2C–E**). In the spleen BCR repertoire, the P_2G10 clonotype was found in 5, 3, and 2 mice in the NOD-FMT, PBS-T, and BCR control groups, respectively, and its mean frequency was likewise over 200- and 900- fold higher than in PBS-T and BCR control mice, respectively. An additional PmE-L–reactive clonotype (P_3D2) was found in the spleen BCR repertoire of one mouse in the NOD-FMT group. Three mouse IgG–reactive clonotypes (G_1H4, G_1E3, and G_1A9) were detected in the spleen BCR repertoire of one, two, and one mice in the NOD-FMT group, respectively. Among these, only the G_1H4 clonotype was also detected in the spleen BCR repertoire of one mouse each in the PBS-T and BCR control groups. Together, these data suggest that stereotypic, autoantigen-reactive BCR clonotypes preferentially expand across NOD-FMT group mice, consistent with a convergent autoimmune B-cell response driven by dysbiotic microbiota transfer.

## 4 Discussion

Previous studies have reported that SjD patients exhibit gut dysbiosis (60–62). Moreover, the transfer and colonization of intestinal microbiota from SjD patients to germ-free mice exacerbated greater corneal barrier disruption after desiccating stress, compared to mice colonized with microbiota from healthy controls (26). The recipient also had decreased levels of CD4^+^ FOXP3^+^ Treg cells in the ocular draining cervical lymph nodes and spleen. To date, however, no study has explored the transplantation of gut microbiota from SjD mouse strain to immunocompetent B6 mice.

The NOD.B10.H2^b^ mouse is a congenic strain carrying the MHC H2^b^ haplotype from the C57BL/10 strain and it develops extensive lymphocytic infiltrates in the exocrine glands (63). Autoimmune dry eye disease spontaneously emerges in this strain by 12 weeks of age (64, 65), and it is therefore frequently used as an SjD animal model (66–69). In a prior study, we demonstrated that modulation of the gut microbiome by probiotics ameliorated disease severity in NOD.B10.H2^b^ mice (69). In the present study, we extended this approach by transferring gut microbiota of NOD.B10.H2^b^ mice to immunocompetent B6 mice, a strain closely related to the C57BL/10 strain (70). FMT is known to induce both local and systemic immune responses. For example, the microbiota of aged B6 mice – enriched with *Bifidobacteria*, *Eubacteria*, and *Akkermansia-*provoked inflammation in the retina and nervous systems after transfer to young mice, whereas FMT from young to aged B6 mice upregulated *Prevotella*, *Lachnospiraceae*, and *Faecalibaculum* populations, and reversed age-associated inflammation (71). Consistently, our study clearly demonstrated that FMT from NOD.B10.H2^b^ mice to B6 mice induced ocular surface inflammation, mirroring findings from human-to-mouse transfer of microbiota from SjD patients (26).

NEI score significantly increased after FMT in the NOD-FMT group, supporting the role of dysbiotic gut microbiota in promoting SjD–like ocular surface inflammation. Unexpectedly, the NEI score significantly decreased after PBS gavage in the PBS-T group (**Figure 2B**). According to previous studies (72, 73), repeated oral gavage may act as a mild procedural stressor in rodents and has been associated with transient stress-related physiological responses, including increased corticosterone levels. Such stress-related systemic immunomodulatory effects may have temporarily influenced ocular surface inflammatory homeostasis and contributed to the transient reduction in fluorescein staining scores observed after PBS gavage. However, ocular surface staining significantly worsened after FMT in NOD-FMT group, suggesting that the pathogenic effects induced by microbiota transfer from SjD–like donor mice outweighed the potential immunomodulatory effects associated with gavage-related stress. These findings further support the role of dysbiotic gut microbiota in promoting SjD–like ocular surface inflammation.

FMT also altered the gut microbiota composition in the NOD-FMT group. Notably, *Blautia* and *Escherichia-Shigella*, which were elevated in SjD patients, were enriched, while *Parasutterella*, previously associated with autoimmunity, was also increased (74).

Mucosal and systemic microbiota exposures are known to shape the B cell repertoire and to regulate inflammatory immune responses in mouse model (75, 76). Here, we examined how gut microbiota transfer from autoimmune dry eye mice to healthy recipient mice impacted the BCR repertoire. Despite the intrinsic diversity of murine BCR repertoires (77), we found that FMT induced a concerted immune response, marked by the emergence of highly stereotypic BCR clonotypes in both the LG and spleen of the NOD-FMT group.

These responses appear to have been triggered by specific antigens in the fecal microbiota of NOD.B10.H2^b^ mice, as the number of stereotypic BCR clonotypes was significantly higher in both the LG and spleen repertoires of the NOD-FMT group, compared to the PBS-T group. In addition, the average frequency of the stereotypic clonotypes in the LG and spleen of the NOD-FMT group was significantly higher than that of the total BCR clonotypes in the corresponding tissues. This pattern suggests clonal expansion of B cells encoding these stereotypic BCRs in both in the LG and spleen, indicating that the transferred microbiota shapes local as well as systemic humoral response.

Humoral immunity has long been implicated in SjD pathogenesis. For instance, immunization with the the Ro60-316–335 peptide in C3H/HeJ or C3H/HeN mice induced SjD-like features, including decreased tear secretion, lymphocyte infiltration into the LGs, antibody production, and elevated cytokine levels (78). Conversely, depletion of B cells with anti-CD20 monoclonal antibodies prevented disease development in C3H/HeN mice. Similarly, in the C57BL/6.NOD-Aec1Aec2 mouse model, B cells were essential for overt SjD-like disease, regardless of T cell infiltration to LGs (79, 80).

The microbiome has also been linked to autoantibody generation and SjD-like clinical features in multiple models. In our prior research, the immunization with *Prevotella melaninogenica* aquaporin-derived peptide induced anti-aquaporin 5 autoantibodies and reduced salivary flow (41). Repeated intraperitoneal injections of *E. coli* induced mononuclear cell infiltration in Harderian and submandibular salivary glands in female C57BL/6 mice (81). And the inflammation was adoptively transferable via splenocytes into immune-deficient Rag2 knockout mice and via CD4^+^ T cells into mature T cell-deficient TCRβ-TCRδ knockout mice. Mass spectrometry analysis identified outer membrane protein A (OmpA) as a key antigen, and repeated injections of recombinant OmpA recapitulated SjD-like features while inducing anti-SSA/Ro and SSB/La autoantibodies (81).

The expansion of stereotypic BCR clonotypes in the LG and spleen of the NOD-FMT group parallels clinical features of SjD patients, where expanded B cell clones dominate the labial salivary glands, with one or two prominent clones present in over 50% of micro-dissected samples (82). Similarly, expanded B−cell clones are found among PB CD21^-^/low B cells, and their somatic hypermutation lineage patterns suggest strong antigen−driven selection. The expanded clones have also been observed in microdissected labial salivary glands and, less frequently, in blood, consistent with bidirectional trafficking between inflamed tissues and the systemic circulation (83).

In an effort to find the FMT-induced autoantibodies, we selected two candidate self-associated antigens relevant to SjD mouse models, PmE-L and mouse IgG, and screened scFv phage display libraries generated from cDNA prepared from the spleens of NOD-FMT mice. This approach successfully identified stereotypic, autoantigen-reactive BCR clonotypes preferentially expand across NOD-FMT group. However, one limitation of our study is that the antigens recognized by majority of these stereotypic BCR clonotypes still remain unidentified. This is largely due to the lack of paired immunoglobulin sequences corresponding to the stereotypic BCR heavy chains. Single cell sequencing of LGs from NOD-FMT mice could address this. However, the abundance of plasma cells in murine LGs has been reported to be below 3% of the total cell population in an SjD model (84). With an average yield of 5, 000 cells per tissue, this translates to fewer than 150 plasma cells per sample, and practical recovery may even lower given the robust immunoglobulin mRNA output of plasma cells (85), which complicates reconstruction of natural heavy-light chain pairs. Future studies that recover paired heavy- and light-chain sequences from these rare plasma cells will therefore be needed to identify the cognate antigens of these stereotypic BCR clonotypes.

Identifying the antigens recognized by these stereotypic BCR clonotypes would provide critical insight into microbiome-driven autoantibody formation. Clinically, the mass spectrometry analysis of immune complexes isolated from the saliva of SjD (n = 9) or xerostomia (n = 7) patients revealed neutrophil defensin 1 (67%), small proline-rich protein 2D (67%), myeloperoxidase (44%), neutrophil elastase (44%), cathepsin G (33%), nuclear mitotic apparatus 1 (33%), and phosphatidylinositol 4-phosphate 3-kinase C2 domain-containing subunit gamma (33%) (86). Several of these antigens share sequence homology with oral microbiota proteins. Immune complex analysis from sera of SjD patients revealed limited overlap with saliva (less than 30%), suggesting predominant local complex formation. The presence of stereotypic autoantigens, their sequence similarity to microbial proteins, and their localization pattern are consistent with our finding that microbiota transfer drives the emergence and local evolution of stereotypic B cell clones.

Finally, as an exploratory, hypothesis-generating investigation, this study did not include *a priori* sample size calculation, as the effect sizes and variability required for such an estimate were not available beforehand; group sizes were instead guided by feasibility and the 3R principle of reduction (87, 88). The relatively small sample size may therefore have limited statistical power to detect more subtle biological and immunological effects, and the present findings should be considered preliminary. Larger, adequately powered studies — incorporating the variability estimates obtained here and functional validation of antigen specificity — will be needed to better characterize microbiota-driven ocular surface inflammation and B-cell responses in SjD.

Our study demonstrated that FMT from an autoimmune dry eye disease model mice induces SjD-like clinicopathological features in immunocompetent B6 mice. In the recipient of FMT, the intestinal microbiome was reshaped in a coordinated manner. Moreover, stereotypic BCR clonotypes emerged and expanded within LGs before ultimately establishing systemic representation.

## Data Availability

The microbiome datasets generated for this study can be found in the NCBI BioProject (accession number: PRJNA1099308; https://www.ncbi.nlm.nih.gov/bioproject/1099308) and the BCR gene sequence datasets in the NCBI GEO (accession number: GSE263577; https://www.ncbi.nlm.nih.gov/geo/query/acc.cgi?acc=GSE263577).
