# Supplementary material for: Gut microbiota transfer from autoimmune dry eye mice imprints stereotypic B cell receptor repertoires in the lacrimal gland and induces disease
Source: Front Immunol. 2026 Jun 16;17:1827057. doi: 10.3389/fimmu.2026.1827057 (PMC13314527; doi:10.3389/fimmu.2026.1827057)
Supplement: Supplementary file 1 [file Image1.pdf]

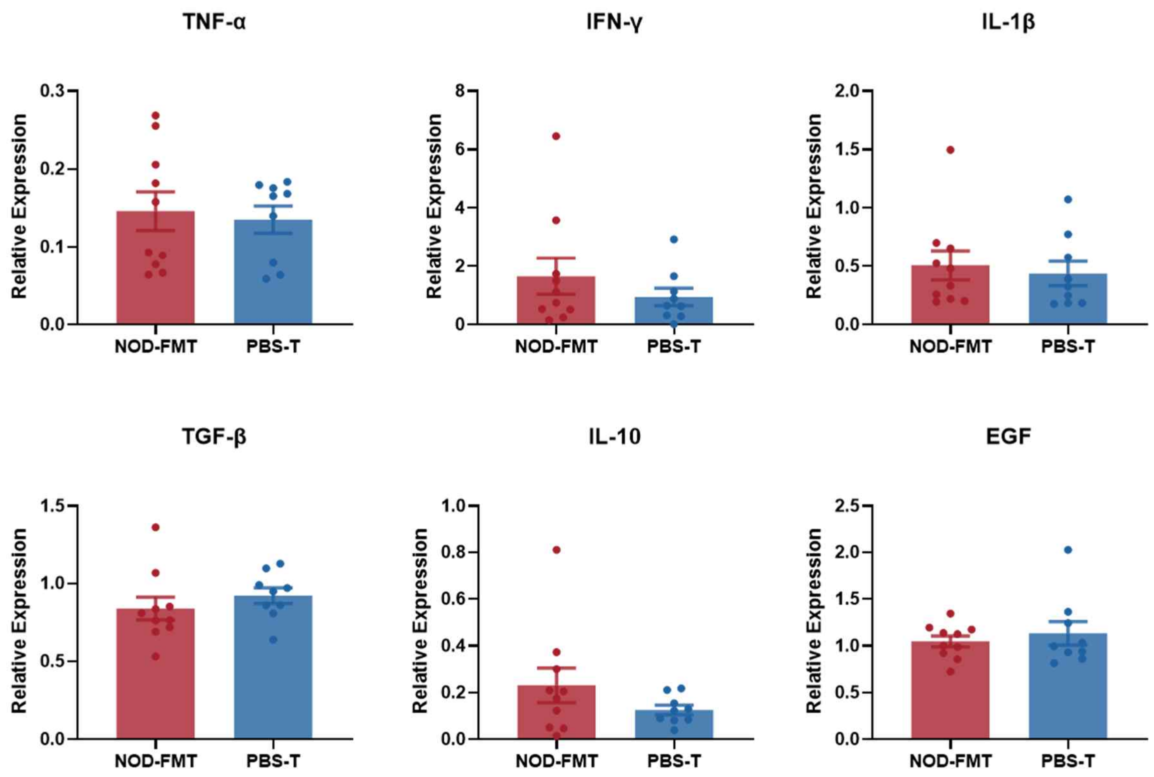

Supplementary Figure 1. mRNA levels of TNF-α, IFN-γ, IL-1β, TGF-β, IL-10 and EGF determined by qRT-PCR
