# Supplementary material for: Gut microbiota transfer from autoimmune dry eye mice imprints stereotypic B cell receptor repertoires in the lacrimal gland and induces disease
Source: Front Immunol. 2026 Jun 16;17:1827057. doi: 10.3389/fimmu.2026.1827057 (PMC13314527; doi:10.3389/fimmu.2026.1827057)
Supplement: Supplementary file 2 [file Image2.pdf]

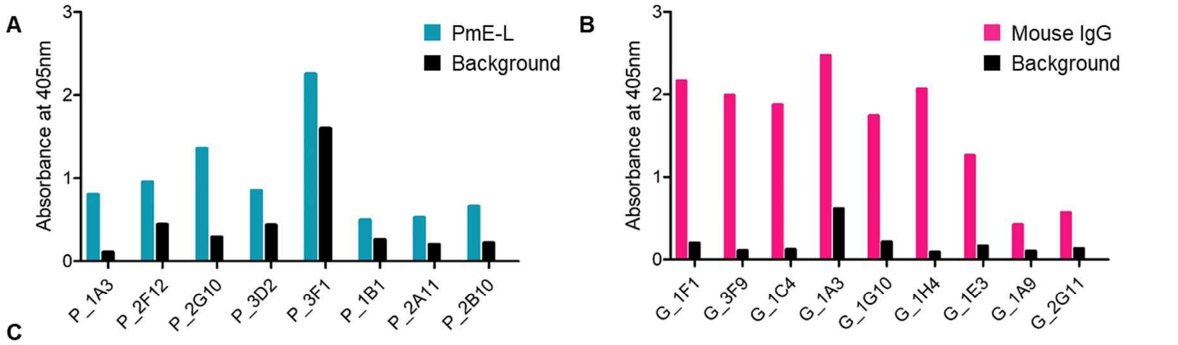

C

Clone_ID	IGHV	IGHJ	HCDR3_AA	IGLV	IGLJ	LCDR3_AA	Degree of sharing (LG)			Degree of sharing (SP)		
							NOD-FMT	PBS-T	BCR control	NOD-FMT	PBS-T	BCR control
P_2G10	IGHV1-9	IGHJ2	ARGGDYFDY	IGLV2	IGLJ2	ALWYSTHFV	**2**	2	-	**5**	3	2
P_3D2	IGHV7-3	IGHJ2	ARQLGRGERDY	IGLV1	IGLJ1	ALWYSNRWV	-	-	-	**1**	-	-
G_1H4	IGHV1-26	IGHJ2	ARRGYDGYDY	IGLV1	IGLJ1	ALWYSNHLV	-	-	-	**1**	1	1
G_1E3	IGHV1-53	IGHJ2	ARLGGSSYYFDY	IGKV3-2	IGKJ1	QQSKEVPWT	-	-	-	**2**	-	-
G_1A9	IGHV1-53	IGHJ2	ARSGGYGNPFDY	IGKV4-58	IGKJ4	HQWSSYPPT	-	-	-	**1**	-	-

D

Clone ID	Mean Frequency (LG)			Mean Frequency (SP)		
	NOD-FMT	PBS-T	BCR control	NOD-FMT	PBS-T	BCR control
P_2G10	**$8.8x10^{-2}$**	$1.3x10^{-4}$	-	**$1.1x10^{-2}$**	$4.1x10^{-5}$	$1.2x10^{-5}$
P_3D2	-	-	-	$9.5x10^{-6}$	-	-
G_1H4	-	-	-	$1.7x10^{-5}$	$1.7x10^{-6}$	$1.8x10^{-6}$
R3_1E3	-	-	-	$7.4x10^{-5}$	-	-
R3_1A9	-	-	-	$1.9x10^{-5}$	-	-

E

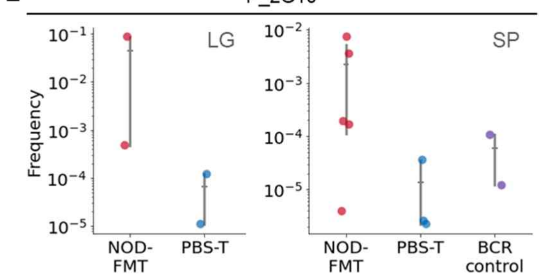

Supplementary Figure 2. Characteristics of NOD-FMT-induced autoreactive BCR clonotypes. (A, B) ELISA screening of PmE-L-reactive **(A)** and mouse IgG-reactive **(B)** clones from an scFv phage display library constructed using cDNA prepared from spleen samples of NOD-FMT mice. Bacterial periplasmic extracts were prepared from individual *E. coli* clones and incubated in antigen-coated wells. The amount of bound antibodies was determined using horseradish peroxidase (HRP)-conjugated anti-HA antibody and ABTS substrate. Background signals were measured from BSA-blocked wells without antigen coating. Bars represent absorbance values for individual clones. **(C)** Stereotypic PmE-L- and mouse IgG-reactive BCR clonotypes shared between scFv clones and the endogenous lacrimal gland (LG) and spleen (SP) BCR repertoires of NOD-FMT, PBS-T, and BCR control group mice, with their encoding V/J genes and CDR3 sequences. **(D)** Mean frequencies of stereotypic clonotypes in LG and SP BCR repertoires. **(E)** Frequency distribution of the P_2G10 clonotype among NOD-FMT, PBS-T, and BCR control group mice. Each dot represents clonotype frequency in an individual mouse from LG or spleen repertoires.
