# Supplementary material for: Gut microbiota transfer from autoimmune dry eye mice imprints stereotypic B cell receptor repertoires in the lacrimal gland and induces disease
Source: Front Immunol. 2026 Jun 16;17:1827057. doi: 10.3389/fimmu.2026.1827057 (PMC13314527; doi:10.3389/fimmu.2026.1827057)
Supplement: Supplementary file 3 [file Table1.docx]

Supplementary Material

**Supplementary Table 1. BCR heavy chain gene-specific primers for NGS analysis**

| **VH** | **Sequence (5’ to 3’)** |
| --- | --- |
| mnphf1 | ACACTCTTTCCCTACACGACGCTCTTCCGATCTCAGGTCCAACTGCAGCAGC |
| mnphf2 | ACACTCTTTCCCTACACGACGCTCTTCCGATCTGAAGTGAAGCTGGTGGAGTCTG |
| mnphf3 | ACACTCTTTCCCTACACGACGCTCTTCCGATCTCAGGTGCAGCTGAAGGAGTC |
| mnphf4 | ACACTCTTTCCCTACACGACGCTCTTCCGATCTCAGATCCAGTTGGTGCAGTCTG |
| mnphf5 | ACACTCTTTCCCTACACGACGCTCTTCCGATCTGAGGTCCAGCTGCAACAGTC |
| mnphf6 | ACACTCTTTCCCTACACGACGCTCTTCCGATCTCAGGTTACTCTGAAAGAGTCTGGC |
| mnphf7 | ACACTCTTTCCCTACACGACGCTCTTCCGATCTGAGGTTCAGCTGCAGCAGTC |
| mnphf8 | ACACTCTTTCCCTACACGACGCTCTTCCGATCTGAGGTGCAGCTTGTTGAGTCTG |
| mnphf9 | ACACTCTTTCCCTACACGACGCTCTTCCGATCTGATGTGCAGCTTCAGGAGTCAG |
| mnphf10 | ACACTCTTTCCCTACACGACGCTCTTCCGATCTGAAGTGCAGCTGTTGGAGACTG |
| mnphf11 | ACACTCTTTCCCTACACGACGCTCTTCCGATCTGAGGTGAAGCTGGTGGAATCTG |
| mnphf12 | ACACTCTTTCCCTACACGACGCTCTTCCGATCTGAGGTCCAGCTGCAACAATCTG |
| mnphf13 | ACACTCTTTCCCTACACGACGCTCTTCCGATCTCAGGTGCAGCTTGTAGAGACC |
| mnphf14 | ACACTCTTTCCCTACACGACGCTCTTCCGATCTCAGATCCAGCTGCAGCAGTC |
| mnphf15 | ACACTCTTTCCCTACACGACGCTCTTCCGATCTCAGGTTCACCTACAACAGTCTGG |
| mnphf16 | ACACTCTTTCCCTACACGACGCTCTTCCGATCTGAGGTCCAGCTGCAACAGTTTG |
| mnphf17 | ACACTCTTTCCCTACACGACGCTCTTCCGATCTGAGGTGAAGCTTCTCGAGTCTG |
| mnphf18 | ACACTCTTTCCCTACACGACGCTCTTCCGATCTGATGTGAACTTGGAAGTGTCTGGAG |
| mnphf19 | ACACTCTTTCCCTACACGACGCTCTTCCGATCTCAGGCTTATCTACAGCAGTCTGG |
| mnphf20 | ACACTCTTTCCCTACACGACGCTCTTCCGATCTCAGCGTGAGCTGCAGCAG |
| mnphf21 | ACACTCTTTCCCTACACGACGCTCTTCCGATCTCAGATTCAGCTTAAGGAGTCTGGAC |
| mnphf22 | ACACTCTTTCCCTACACGACGCTCTTCCGATCTCAGGTGCAGATGAAGCAGTCAG |
| mnphf23 | ACACTCTTTCCCTACACGACGCTCTTCCGATCTCAGGTGCAGAATAAGTCAGGACC |
| mnphf24 | ACACTCTTTCCCTACACGACGCTCTTCCGATCTCAAGTGCAGATGAAGGAGTCAGG |
| mnphf25 | ACACTCTTTCCCTACACGACGCTCTTCCGATCTGATGTGCAGCTTCAGGAGTCG |
| mnphf26 | ACACTCTTTCCCTACACGACGCTCTTCCGATCTGATGTGCAGCTGGTGGAGTC |
| mnphf27 | ACACTCTTTCCCTACACGACGCTCTTCCGATCTGACGTGAAGCTCGTGGAGTC |
| mnphf28 | ACACTCTTTCCCTACACGACGCTCTTCCGATCTGAGGAGAAGCTGGATGAGTCTGG |
| mnphf29 | ACACTCTTTCCCTACACGACGCTCTTCCGATCTCAGGTCCAGCTGCAGCAG |
| mnphf30 | ACACTCTTTCCCTACACGACGCTCTTCCGATCTGAGGTGAAGCTTGAGGAGTCTG |
| mnphf31 | ACACTCTTTCCCTACACGACGCTCTTCCGATCTCAGATGCAGCTTCAGGAGTCAG |
| mnphf32 | ACACTCTTTCCCTACACGACGCTCTTCCGATCTCAGGTCCAGCTGAAGCAGTC |
| mnphf33 | ACACTCTTTCCCTACACGACGCTCTTCCGATCTTGGTATCAACGCAGAGCTTGG |
| mnphf34 | ACACTCTTTCCCTACACGACGCTCTTCCGATCTGAAGTGAAACTTGAGGAGTCTGGAG |
| mnphf35 | ACACTCTTTCCCTACACGACGCTCTTCCGATCTGAAGTGAAGTTTGAGGAGTCTGGAG |
| **CH1** | **Sequence (5’ to 3’)** |
| mnhr1_m | TGACTGGAGTTCAGACGTGTGCTCTTCCGATCTNNNNTNNNNTNNNNAGACGAGGGGGAAGACATTTGG |
| mnhr2_d | TGACTGGAGTTCAGACGTGTGCTCTTCCGATCTNNNNTNNNNTNNNNTCTGAGAGGAGGAACATGTCAGG |
| mnhr3_g | TGACTGGAGTTCAGACGTGTGCTCTTCCGATCTNNNNTNNNNTNNNNCCAGGGGCCAGTGGATAGAC |
| mnhr4_g | TGACTGGAGTTCAGACGTGTGCTCTTCCGATCTNNNNTNNNNTNNNNACAGGGGCCAGTGGATAGAC |
| mnhr5_g | TGACTGGAGTTCAGACGTGTGCTCTTCCGATCTNNNNTNNNNTNNNNGCCAGGGACCAAGGGATAGAC |
| mnhr6_a | TGACTGGAGTTCAGACGTGTGCTCTTCCGATCTNNNNTNNNNTNNNNTGTCAGTGGGTAGATGGTGGG |
| mnhr7_e | TGACTGGAGTTCAGACGTGTGCTCTTCCGATCTNNNNTNNNNTNNNNGGCTTTAAGGGGTAGAGCTGAGG |
| **Indexing** | **Sequence (5’ to 3’)** |
| IA5_AMP | AATGATACGGCGACCACCGAGATCTACAC[i5 index]ACACTCTTTCCCTACACGACGCTCTTCCGATC |
| IA7_AMP | CAAGCAGAAGACGGCATACGAGAT[i7 index]GTGACTGGAGTTCAGACGTGTGCTCTTCCG |

**Supplementary Table 2. Statistics for the taxa with LFC scores >2**

| **Taxa** | **LFC** | **p-value** | **q-value** |
| --- | --- | --- | --- |
| *Uncultured_order_Rhodospirillales* | 6.7610 | 2.49E-20 | 2.17E-18 |
| *Blautia* | 6.2741 | 7.98E-14 | 6.07E-12 |
| *Alloprevotella* | 5.3523 | 2.03E-18 | 1.69E-16 |
| *Parasutterella* | 5.3060 | 7.11E-101 | 6.83E-99 |
| *Tannerellaceae* | 4.9381 | 3.78E-19 | 3.25E-17 |
| *Desulfovibrio* | 4.1884 | 1.57E-17 | 1.27E-15 |
| *Helicobacter* | 3.9507 | 2.19E-16 | 1.69E-14 |
| *Escherichia-Shigella* | 3.9073 | 5.81E-05 | 3.43E-03 |
| *Lactobacillus* | 2.7182 | 4.11E-09 | 2.80E-07 |
| *Unclassified_family_Prevotellaceae* | 2.4778 | 3.39E-03 | 1.43E-01 |
| *Unclassified_order_Enterobacterales* | 2.4514 | 8.45E-04 | 4.31E-02 |
| *Rikenella* | 2.3171 | 6.11E-17 | 4.83E-15 |
| *Enterococcus* | 2.2983 | 3.75E-03 | 1.54E-01 |

**Supplementary Table 3. Statistics for the processing of the lacrimal gland IGH NGS data in NOD-FMT, PBS-T, and BCR control groups.**

| **Sample** | **Organ** | **Raw reads** | **Unique consensus sequence reads** | **Unique consensus sequence reads after contamination elimination** | **Clonotypes** |
| --- | --- | --- | --- | --- | --- |
| NOD-FMT1 | Lacrimal gland | 4,865,268 | 243,954 | 243,863 | 1,946 |
| NOD-FMT2 | Lacrimal gland | 2,346,953 | 264,744 | 264,692 | 1,672 |
| NOD-FMT3 | Lacrimal gland | 6,083,941 | 62,433 | 62,316 | 702 |
| NOD-FMT4 | Lacrimal gland | 5,841,778 | 61,479 | 61,344 | 770 |
| NOD-FMT5 | Lacrimal gland | 1,583,739 | 154,470 | 154,456 | 732 |
| NOD-FMT6 | Lacrimal gland | 4,542,199 | 54,009 | 53,757 | 814 |
| NOD-FMT7 | Lacrimal gland | 4,814,990 | 341,412 | 341,343 | 2,489 |
| NOD-FMT8 | Lacrimal gland | 4,023,242 | 97,080 | 97,052 | 708 |
| NOD-FMT9 | Lacrimal gland | 2,314,443 | 173,863 | 173,797 | 1,259 |
| NOD-FMT10 | Lacrimal gland | 2,206,919 | 175,280 | 175,238 | 1,157 |
| PBS-T1 | Lacrimal gland | 3,056,835 | 42,241 | 42,097 | 807 |
| PBS-T2 | Lacrimal gland | 2,668,168 | 90,238 | 90,197 | 553 |
| PBS-T3 | Lacrimal gland | 3,538,196 | 40,513 | 40,419 | 812 |
| PBS-T4 | Lacrimal gland | 2,246,717 | 57,793 | 57,776 | 619 |
| PBS-T5 | Lacrimal gland | 5,626,990 | 360,216 | 360,072 | 17,186 |
| PBS-T6 | Lacrimal gland | 3,273,104 | 56,812 | 56,726 | 649 |
| PBS-T7 | Lacrimal gland | 2,950,861 | 50,972 | 50,473 | 447 |
| PBS-T8 | Lacrimal gland | 2,787,600 | 81,331 | 81,136 | 679 |
| PBS-T9 | Lacrimal gland | 2,499,731 | 113,692 | 113,543 | 939 |
| BCR control 1 | Lacrimal gland | 3,162,240 | 80,834 | 80,510 | 818 |
| BCR control 2 | Lacrimal gland | 3,218,558 | 64,963 | 61,573 | 594 |
| BCR control 3 | Lacrimal gland | 2,720,763 | 77,100 | 76,494 | 909 |
| BCR control 4 | Lacrimal gland | 6,077,877 | 57,398 | 56,884 | 854 |
| BCR control 5 | Lacrimal gland | 2,299,433 | 75,590 | 75,290 | 781 |

**Supplementary Table 4. Statistics for the processing of the spleen IGH NGS data in NOD-FMT, PBS-T, and BCR control groups.**

| **Sample** | **Organ** | **Raw reads** | **Unique consensus sequence reads** | **Unique consensus sequence reads after contamination elimination** | **Clonotypes** |
| --- | --- | --- | --- | --- | --- |
| NOD-FMT1 | Spleen | 6,560,986 | 512,399 | 495,663 | 184,443 |
| NOD-FMT2 | Spleen | 5,023,465 | 168,907 | 162,996 | 66,217 |
| NOD-FMT3 | Spleen | 33,194,276 | 2,302,185 | 2,280,764 | 586,356 |
| NOD-FMT4 | Spleen | 3,862,004 | 358,350 | 345,811 | 122,078 |
| NOD-FMT5 | Spleen | 4,644,796 | 447,466 | 438,384 | 122,735 |
| NOD-FMT6 | Spleen | 2,103,631 | 269,360 | 261,669 | 77,024 |
| NOD-FMT7 | Spleen | 9,621,912 | 593,110 | 583,413 | 158,723 |
| NOD-FMT8 | Spleen | 6,586,581 | 462,252 | 448,566 | 135,626 |
| NOD-FMT9 | Spleen | 7,191,385 | 694,861 | 680,374 | 179,616 |
| NOD-FMT10 | Spleen | 7,109,060 | 421,938 | 409,684 | 145,558 |
| PBS-T1 | Spleen | 4,109,406 | 389,289 | 379,717 | 102,983 |
| PBS-T2 | Spleen | 3,396,450 | 270,480 | 264,045 | 58,537 |
| PBS-T3 | Spleen | 4,258,139 | 281,952 | 274,147 | 103,991 |
| PBS-T4 | Spleen | 8,459,727 | 575,391 | 559,050 | 195,924 |
| PBS-T5 | Spleen | 3,433,385 | 390,247 | 381,045 | 128,217 |
| PBS-T6 | Spleen | 3,674,747 | 215,159 | 213,063 | 22,478 |
| PBS-T7 | Spleen | 5,800,271 | 237,050 | 231,280 | 94,006 |
| PBS-T8 | Spleen | 8,944,910 | 591,813 | 582,840 | 123,303 |
| PBS-T9 | Spleen | 6,351,394 | 447,785 | 437,700 | 96,006 |
| BCR control 1 | Spleen | 8,950,950 | 570,376 | 553,094 | 186,977 |
| BCR control 2 | Spleen | 2,870,651 | 86,717 | 83,079 | 40,240 |
| BCR control 3 | Spleen | 6,132,458 | 417,470 | 406,147 | 133,900 |
| BCR control 4 | Spleen | 3,879,320 | 377,894 | 369,647 | 142,294 |
| BCR control 5 | Spleen | 6,826,603 | 584,159 | 575,030 | 183,895 |

**Supplementary Table 5. P-values from Mann-Whitney tests on the degree of sharing of stereotypic clonotypes for each group**

| **Group** | **Organ** | **p-value** |
| --- | --- | --- |
| NOD-FMT vs PBS-T | Lacrimal gland | <0.0001  (5.07e-6) |
|  | Spleen | <0.0001  (2.19e-37) |

**Supplementary Table 6. Statistical analysis on the frequency of all clonotypes and stereotypic clonotypes**

| **Group** | **Organ** | **All** | | **Stereotypic** | | **p-value** | **Top %** |
| --- | --- | --- | --- | --- | --- | --- | --- |
|  |  | **Freq avg** | **Freq std** | **Freq avg** | **Freq std** |  |  |
| NOD-FMT | Lacrimal gland | 8.05e-4 | 5.97e-3 | 4.78e-3 | 2.22e-2 | <0.0001 | 3.28% |
| PBS-T |  | 3.63e-4 | 4.44e-3 | 2.66e-3 | 1.80e-3 | <0.0001 | 2.08% |
| NOD-FMT | Spleen | 4.69e-6 | 1.11e-4 | 1.52e-5 | 1.31e-4 | <0.0001 | 2.56% |
| PBS-T |  | 8.48e-6 | 8.76e-4 | 2.74e-5 | 3.29e-4 | <0.0001 | 2.36% |

**Supplementary Table 7. Statistical analysis for the number of germline-containing clonotypes among lacrimal gland stereotypic clonotypes**

| **Group** | **Number of lacrimal gland**  **stereotypic clonotypes** | | **p-value** | **Cramer’s V** |
| --- | --- | --- | --- | --- |
|  | **Without germline  (SHM=0) sequences** | **With germline  (SHM=0) sequences** |  |  |
| NOD-FMT | 30 | 157 | 3.94e-6 | 0.25 |
| PBS-T | 57 | 89 |  |  |

**Supplementary Table 8. Statistical analysis for the number of germline-containing clonotypes among spleen stereotypic clonotypes**

| **Group** | **Number of spleen**  **stereotypic clonotypes** | | **p-value** | **Cramer’s V** |
| --- | --- | --- | --- | --- |
|  | **Without germline  (SHM=0) sequences** | **With germline  (SHM=0) sequences** |  |  |
| NOD-FMT | 173 | 33,567 | 0.708 | 0.002 |
| PBS-T | 50 | 9,014 |  |  |

**Supplementary Table 9. Statistical analysis for the number of lacrimal gland stereotypic clonotypes found in spleen**

| **Group** | **Number of lacrimal gland**  **stereotypic clonotypes** | | **p-value** | **Cramer’s V** |
| --- | --- | --- | --- | --- |
|  | **Without germline  (SHM=0) sequences** | **With germline  (SHM=0) sequences** |  |  |
| NOD-FMT | 123 | 64 | < 0.0001  (2.08e-13) | 0.402 |
| PBS-T | 36 | 110 |  |  |

**Supplementary Table 10. Statistical analysis for the number of spleen stereotypic clonotypes found in lacrimal gland**

| **Group** | **Number of spleen**  **stereotypic clonotypes** | | **p-value** | **Cramer’s V** |
| --- | --- | --- | --- | --- |
|  | **Found in**  **lacrimal gland** | **Not found in lacrimal gland** |  |  |
| NOD-FMT | 284 | 34,740 | 0.121 | 0.007 |
| PBS-T | 90 | 9,064 |  |  |

**Supplementary Table 11. Statistics for the processing of the PB IGH NGS data in NOD-FMT, PBS-T, and BCR control groups.**

| **Sample** | **Organ** | **Raw reads** | **Unique consensus sequence reads** | **Unique consensus sequence reads after contamination elimination** | **Clonotypes** |
| --- | --- | --- | --- | --- | --- |
| NOD-FMT | PB | 2,395,222 | 251,200 | 246,351 | 202,436 |
| PBS-T | PB | 2,255,563 | 200,819 | 196,363 | 168,763 |

**Supplementary Table 12. Number of LG stereotypic BCR clonotypes found in the pooled PB**

| **Group** | **Degree of sharing** | | | | | | | | | **Total** |
| --- | --- | --- | --- | --- | --- | --- | --- | --- | --- | --- |
|  | **2** | **3** | **4** | **5** | **6** | **7** | **8** | **9** | **10** |  |
| NOD-FMT | 34/153  (22.2%) | 14/27  (51.6%) | 1/3  (33.3%) | 3/3  (100%) | - | - | - | 1/1  (100%) | - | 53/187 (28.3 %) |
| PBS-T | 25/142 (17.6%) | 2/4 (50.0 %) | - | - | - | - | - | - | **-** | 27/146 (18.5 %) |

**Supplementary Table 13. Statistical analysis for the number of lacrimal gland stereotypic clonotypes found in PB**

| **Group** | **Number of lacrimal gland**  **stereotypic clonotypes** | | **p-value** | **Cramer’s V** |
| --- | --- | --- | --- | --- |
|  | **Found in**  **PB** | **Not found in**  **PB** |  |  |
| NOD-FMT | 53 | 134 | 0.050 | 0.107 |
| PBS-T | 27 | 119 |  |  |

**Supplementary Table 14. Number of spleen stereotypic BCR clonotypes found in the pooled PB**

| **Group** | **Degree of sharing** | | | | | | | | | **Total** |
| --- | --- | --- | --- | --- | --- | --- | --- | --- | --- | --- |
|  | **2** | **3** | **4** | **5** | **6** | **7** | **8** | **9** | **10** |  |
| NOD-FMT | 1773/30279  (5.9%) | 300/2922  (10.3%) | 72/415  (17.3%) | 23/82  (28.0%) | 14/29  (48.3%) | 4/6  (66.7%) | 5/5  (100%) | 1/1  (100%) | 1/1  (100%) | 2193/33740  (6.5%) |
| PBS-T | 468/8527  (5.5%) | 47/496  (9.5%) | 8/39  (20.5%) | 1/2  (50%) | - | - | - | - | - | 524/9064  (5.8%) |

**Supplementary Table 15. Statistical analysis for the number of spleen stereotypic clonotypes found in PB**

| **Group** | **Number of spleen**  **stereotypic clonotypes** | | **p-value** | **Cramer’s V** |
| --- | --- | --- | --- | --- |
|  | **Found in**  **PB** | **Not found in PB** |  |  |
| NOD-FMT | 2,193 | 32,547 | 0.065 | 0.009 |
| PBS-T | 524 | 8,540 |  |  |

**Supplementary Table 16. Primers for scFv phage display library construction**

| **Heavy chain** | | |
| --- | --- | --- |
| **V gene** | | **Sequence (5’ to 3’)** |
| P mhf1 | | GGTGGTTCCTCTAGATCTTCCCTCCAGGTCCAACTGCAGCAGC |
| P_mhf2 | | GGTGGTTCCTCTAGATCTTCCCTCGAAGTGAAGCTGGTGGAGTCTG |
| P_mhf3 | | GGTGGTTCCTCTAGATCTTCCCTCCAGGTGCAGCTGAAGGAGTC |
| P_mhf4 | | GGTGGTTCCTCTAGATCTTCCCTCCAGATCCAGTTGGTGCAGTCTG |
| P_mhf5 | | GGTGGTTCCTCTAGATCTTCCCTCGAGGTCCAGCTGCAACAGTC |
| P_mhf6 | | GGTGGTTCCTCTAGATCTTCCCTCCAGGTTACTCTGAAAGAGTCTGGC |
| P_mhf7 | | GGTGGTTCCTCTAGATCTTCCCTCGAGGTTCAGCTGCAGCAGTC |
| P_mhf8 | | GGTGGTTCCTCTAGATCTTCCCTCGAGGTGCAGCTTGTTGAGTCTG |
| P_mhf9 | | GGTGGTTCCTCTAGATCTTCCCTCGATGTGCAGCTTCAGGAGTCAG |
| P_mhf10 | | GGTGGTTCCTCTAGATCTTCCCTCGAAGTGCAGCTGTTGGAGACTG |
| P_mhf11 | | GGTGGTTCCTCTAGATCTTCCCTCGAGGTGAAGCTGGTGGAATCTG |
| P_mhf12 | | GGTGGTTCCTCTAGATCTTCCCTCGAGGTCCAGCTGCAACAATCTG |
| P_mhf13 | | GGTGGTTCCTCTAGATCTTCCCTCCAGGTGCAGCTTGTAGAGACC |
| P_mhf14 | | GGTGGTTCCTCTAGATCTTCCCTCCAGATCCAGCTGCAGCAGTC |
| P_mhf15 | | GGTGGTTCCTCTAGATCTTCCCTCCAGGTTCACCTACAACAGTCTGG |
| P_mhf16 | | GGTGGTTCCTCTAGATCTTCCCTCGAGGTCCAGCTGCAACAGTTTG |
| P_mhf17 | | GGTGGTTCCTCTAGATCTTCCCTCGAGGTGAAGCTTCTCGAGTCTG |
| P_mhf18 | | GGTGGTTCCTCTAGATCTTCCCTCGATGTGAACTTGGAAGTGTCTGGAG |
| P_mhf19 | | GGTGGTTCCTCTAGATCTTCCCTCCAGGCTTATCTACAGCAGTCTGG |
| P_mhf20 | | GGTGGTTCCTCTAGATCTTCCCTCCAGCGTGAGCTGCAGCAG |
| P_mhf21 | | GGTGGTTCCTCTAGATCTTCCCTCCAGATTCAGCTTAAGGAGTCTGGAC |
| P_mhf22 | | GGTGGTTCCTCTAGATCTTCCCTCCAGGTGCAGATGAAGCAGTCAG |
| P_mhf23 | | GGTGGTTCCTCTAGATCTTCCCTCCAGGTGCAGAATAAGTCAGGACC |
| P_mhf24 | | GGTGGTTCCTCTAGATCTTCCCTCCAAGTGCAGATGAAGGAGTCAGG |
| P_mhf25 | | GGTGGTTCCTCTAGATCTTCCCTCGATGTGCAGCTTCAGGAGTCG |
| P_mhf26 | | GGTGGTTCCTCTAGATCTTCCCTCGATGTGCAGCTGGTGGAGTC |
| P_mhf27 | | GGTGGTTCCTCTAGATCTTCCCTCGACGTGAAGCTCGTGGAGTC |
| P_mhf28 | | GGTGGTTCCTCTAGATCTTCCCTCGAGGAGAAGCTGGATGAGTCTGG |
| P_mhf29 | | GGTGGTTCCTCTAGATCTTCCCTCCAGGTCCAGCTGCAGCAG |
| P_mhf30 | | GGTGGTTCCTCTAGATCTTCCCTCGAGGTGAAGCTTGAGGAGTCTG |
| P_mhf31 | | GGTGGTTCCTCTAGATCTTCCCTCCAGATGCAGCTTCAGGAGTCAG |
| P_mhf32 | | GGTGGTTCCTCTAGATCTTCCCTCCAGGTCCAGCTGAAGCAGTC |
| P_mhf33 | | GGTGGTTCCTCTAGATCTTCCCTCTGGTATCAACGCAGAGCTTGG |
| P_mhf34 | | GGTGGTTCCTCTAGATCTTCCCTCGAAGTGAAACTTGAGGAGTCTGGAG |
| P_mhf35 | | GGTGGTTCCTCTAGATCTTCCCTCGAAGTGAAGTTTGAGGAGTCTGGAG |
| **J gene** | | **Sequence (5’ to 3’)** |
| P_mhr1 | | CCTGGCCGGCCTGGCCACTAGTTGAGGAGACGGTGACCGTG |
| P_mhr2 | | CCTGGCCGGCCTGGCCACTAGTTGAGGAGACTGTGAGAGTGGTG |
| P_mhr3 | | CCTGGCCGGCCTGGCCACTAGTTGAGGAGACTGTGAGAGAGGTG |
| P_mhr4 | | CCTGGCCGGCCTGGCCACTAGTTGAGGAGACTGTGAGACTGGTG |
| P_mhr5 | | CCTGGCCGGCCTGGCCACTAGTTGCAGAGACAGTGACCAGAGTC |
| P_mhr6 | | CCTGGCCGGCCTGGCCACTAGTTGAGGAGACGGTGACTGAGG |
| **Light chain Kappa** | | |
| **V gene** | **Sequence (5’ to 3’)** | |
| P_mkf1 | GGGCCCAGGCGGCCGAGCTCGATATCCAGATGACACAGACTACATCC | |
| P_mkf2 | GGGCCCAGGCGGCCGAGCTCCAAATTGTTCTCACCCAGTCTCCAG | |
| P_mkf3 | GGGCCCAGGCGGCCGAGCTCGACATCCAGATGACTCAGTCTCCAG | |
| P_mkf4 | GGGCCCAGGCGGCCGAGCTCGATATTGTGATGACGCAGGCTGC | |
| P_mkf5 | GGGCCCAGGCGGCCGAGCTCGACATTGTGCTGACCCAATCTCC | |
| P_mkf6 | GGGCCCAGGCGGCCGAGCTCAGTATTGTGATGACCCAGACTCCC | |
| P_mkf7 | GGGCCCAGGCGGCCGAGCTCGACATTGTGATGACACAGTCTCCATC | |
| P_mkf8 | GGGCCCAGGCGGCCGAGCTCGATGTTGTGATGACCCAAACTCCAC | |
| P_mkf9 | GGGCCCAGGCGGCCGAGCTCGACATTGTGATGACCCAGTCTCA | |
| P_mkf10 | GGGCCCAGGCGGCCGAGCTCGACATCCAGATGACACAATCTTCATCC | |
| P_mkf11 | GGGCCCAGGCGGCCGAGCTCGAAACAACTGTGACCCAGTCTCC | |
| P_mkf12 | GGGCCCAGGCGGCCGAGCTCGATATTGTGATAACCCAGGATGAACTCTC | |
| P_mkf13 | GGGCCCAGGCGGCCGAGCTCGATATTGTGCTAACTCAGTCTCCAGC | |
| P_mkf14 | GGGCCCAGGCGGCCGAGCTCGACATCCAGATGACCCAGTCTCC | |
| P_mkf15 | GGGCCCAGGCGGCCGAGCTCGATGTCCAGATGATTCAGTCTCCATC | |
| P_mkf16 | GGGCCCAGGCGGCCGAGCTCGACATCCTGATGACCCAATCTCC | |
| P_mkf17 | GGGCCCAGGCGGCCGAGCTCGATGTCCAGATAACCCAGTCTCCATC | |
| P_mkf18 | GGGCCCAGGCGGCCGAGCTCACTGGAGAAACAACACAGGCTC | |
| P_mkf19 | GGGCCCAGGCGGCCGAGCTCAATATCCAGGTGATCCAGTCACCA | |
| P_mkf20 | GGGCCCAGGCGGCCGAGCTCGACATTGTGCTAACACAGTCTCCTG | |
| P_mkf21 | GGGCCCAGGCGGCCGAGCTCGAAAATGTGCTGACCCAGTCTCC | |
| P_mkf22 | GGGCCCAGGCGGCCGAGCTCGACATTGTGATGACTCAGTCTCCAG | |
| P_mkf23 | GGGCCCAGGCGGCCGAGCTCGACATCTTGCTGACTCAGTCTCC | |
| P_mkf24 | GGGCCCAGGCGGCCGAGCTCAGCATTGTGATGACCCAGTCTC | |
| P_mkf25 | GGGCCCAGGCGGCCGAGCTCGAAATTGTGTTGACCCAGTCTATACCATC | |
| **J gene** | **Sequence (5’ to 3’)** | |
| P_mkr1 | GGAAGATCTAGAGGAACCACCCCCACCACCGCCCGAGCCACCGCCACCAGAGGATTTGATTTCCAGCTTGGTGCCTC | |
| P_mkr2 | GGAAGATCTAGAGGAACCACCCCCACCACCGCCCGAGCCACCGCCACCAGAGGATTTTATTTCCAGCTTGGTCCCCC | |
| P_mkr3 | GGAAGATCTAGAGGAACCACCCCCACCACCGCCCGAGCCACCGCCACCAGAGGATTTCATTTCCAGCTTGGTCCCC | |
| P_mkr4 | GGAAGATCTAGAGGAACCACCCCCACCACCGCCCGAGCCACCGCCACCAGAGGATTTTATTTCCAACTTTGTCCCCGAGC | |
| P_mkr5 | GGAAGATCTAGAGGAACCACCCCCACCACCGCCCGAGCCACCGCCACCAGAGGATTTTATTTCCAATTTTGTCCCCGTGC | |
| P_mkr6 | GGAAGATCTAGAGGAACCACCCCCACCACCGCCCGAGCCACCGCCACCAGAGGATTTCAGCTCCAGCTTGGTCC | |
| **Light chain Lambda** | | |
| **V gene** | **Sequence (5’ to 3’)** | |
| P_mlf1 | GGGCCCAGGCGGCCGAGCTCGATCAGGCTGTTGTGACTCAGGAATC | |
| P_mlf2 | GGGCCCAGGCGGCCGAGCTCGATCAACTTGTGCTCACTCAGTCATCTTC | |
| **J gene** | **Sequence (5’ to 3’)** | |
| P_mlr1 | GGAAGATCTAGAGGAACCACCCCCACCACCGCCCGAGCCACCGCCACCAGAGGAGCCTAGGACAGTCAGTTTGGTTCCTCC | |
| P_mlr2 | GGAAGATCTAGAGGAACCACCCCCACCACCGCCCGAGCCACCGCCACCAGAGGAGCCTAGGACAGTGACCTTGGTTCCAC | |
| **Overlap-extension PCR Primer Sequence (5’ to 3’)** | | |
| OVP-F | GGGCCCAGGCGGCCGAG | |
| OVP-R | CCTGGCCGGCCTGGCCA | |
